# Structural Basis for the Inhibition of Histone Deacetylase 8 (HDAC8), a Key Epigenetic Player in the Blood Fluke *Schistosoma mansoni*

**DOI:** 10.1371/journal.ppat.1003645

**Published:** 2013-09-26

**Authors:** Martin Marek, Srinivasaraghavan Kannan, Alexander-Thomas Hauser, Marina Moraes Mourão, Stéphanie Caby, Vincent Cura, Diana A. Stolfa, Karin Schmidtkunz, Julien Lancelot, Luiza Andrade, Jean-Paul Renaud, Guilherme Oliveira, Wolfgang Sippl, Manfred Jung, Jean Cavarelli, Raymond J. Pierce, Christophe Romier

**Affiliations:** 1 Département de Biologie Structurale Intégrative, Institut de Génétique et Biologie Moléculaire et Cellulaire (IGBMC), Université de Strasbourg (UDS), CNRS, INSERM, Illkirch, France; 2 Institut für Pharmazie, Martin-Luther-Universität Halle-Wittenberg, Halle, Germany; 3 Institut für Pharmazeutische Wissenschaften, Albert-Ludwigs-Universität Freiburg, Freiburg, Germany; 4 Genomics and Computational Biology Group, Center for Excellence in Bioinformatics, National Institute of Science and Technology in Tropical Diseases, Centro de Pesquisas René Rachou, Fundação Oswaldo Cruz, Belo Horizonte, Minas Gerais, Brazil; 5 Center for Infection and Immunity of Lille (CIIL), INSERM U1019 – CNRS UMR 8204, Université Lille Nord de France, Institut Pasteur de Lille, Lille, France; 6 Freiburg Institute of Advanced Studies (FRIAS), Albert-Ludwigs-Universität Freiburg, Freiburg, Germany; McGill University, Canada

## Abstract

The treatment of schistosomiasis, a disease caused by blood flukes parasites of the *Schistosoma* genus, depends on the intensive use of a single drug, praziquantel, which increases the likelihood of the development of drug-resistant parasite strains and renders the search for new drugs a strategic priority. Currently, inhibitors of human epigenetic enzymes are actively investigated as novel anti-cancer drugs and have the potential to be used as new anti-parasitic agents. Here, we report that *Schistosoma mansoni* histone deacetylase 8 (smHDAC8), the most expressed class I HDAC isotype in this organism, is a functional acetyl-L-lysine deacetylase that plays an important role in parasite infectivity. The crystal structure of smHDAC8 shows that this enzyme adopts a canonical α/β HDAC fold, with specific solvent exposed loops corresponding to insertions in the schistosome HDAC8 sequence. Importantly, structures of smHDAC8 in complex with generic HDAC inhibitors revealed specific structural changes in the smHDAC8 active site that cannot be accommodated by human HDACs. Using a structure-based approach, we identified several small-molecule inhibitors that build on these specificities. These molecules exhibit an inhibitory effect on smHDAC8 but show reduced affinity for human HDACs. Crucially, we show that a newly identified smHDAC8 inhibitor has the capacity to induce apoptosis and mortality in schistosomes. Taken together, our biological and structural findings define the framework for the rational design of small-molecule inhibitors specifically interfering with schistosome epigenetic mechanisms, and further support an anti-parasitic epigenome targeting strategy to treat neglected diseases caused by eukaryotic pathogens.

## Author Summary

Schistosomiasis, a neglected parasitic disease caused by flatworms of the genus *Schistosoma*, is responsible for hundreds of thousands of deaths yearly. Its treatment currently depends on a single drug, praziquantel, with reports of drug-resistant parasites. Human epigenetic enzymes, in particular histone deacetylases (HDACs), are predominantly attractive inhibitory targets for anti-cancer therapies. Validated scaffolds against these enzymes could also be used as leads in the search for novel specific drugs against schistosomiasis. In our study, we show that *Schistosoma mansoni* histone deacetylase 8 (smHDAC8) is a functional acetyl-L-lysine deacetylase that plays an important role in parasite infectivity and is therefore a relevant target for drug discovery. The determination of the atomic structures of smHDAC8 in complex with generic HDAC inhibitors revealed that the architecture of the smHDAC8 active site pocket differed significantly from its human counterparts and provided a framework for the development of inhibitors selectively interfering with schistosome epigenetic mechanisms. In agreement, this information enabled us to identify several small-molecule scaffolds that possess specific inhibitory effects on smHDAC8 and cause mortality in schistosomes. Our results provide the proof of concept that targeting epigenetic enzymes is a valid approach to treat neglected diseases caused by eukaryotic pathogens.

## Introduction

The need for new drugs against eukaryotic parasites is acute, notably for the neglected parasitic diseases [1] for which no effective vaccines have yet been developed and for which the limited number of drugs available for treatment makes increasingly likely the selection of resistant parasite strains [2]–[5]. This is the case for schistosomiasis (bilharzia), one of the major human neglected parasitic diseases, which is caused by platyhelminth parasites from the genus *Schistosoma* (*S. mansoni*, *S. japonicum*, *S. haematobium*, *S. intercalatum*, *S. mekongi*) [6], [7]. Schistosomes infect around 200 million people worldwide and cause at least 300.000 deaths yearly, with about 800 million people further at risk of infection [8]. The dependence of the control of schistosomiasis on mass treatment with a single drug, praziquantel [9], and the consequent risk of the appearance of resistant strains raises the spectre of widespread drug resistance [10]–[13].

Epigenetic players are increasingly reported to be involved in cancer genesis and progression [14]–[16], which explains the intense targeting of the human epigenome to develop anti-cancer therapies [17]–[22]. Interestingly, many human parasites share several characteristics with malignant tumors, including high metabolic activity, a dependence on lactate fermentation as an energy source within the human host, uncontrolled (by the host) cell division, and a degree of invisibility to the host immune responses [23]. It is therefore expected that an approach targeting parasitic epigenomes would be successful in treating human parasitic diseases caused by eukaryotic organisms.

To speed up the search for novel anti-parasitic drugs, a “piggyback” strategy can be used that builds on chemical scaffolds previously validated for other diseases [24]. In humans, histone deacetylases (HDACs) are among the most studied epigenetic targets [25], and a variety of HDAC inhibitors affecting cancer cells have been discovered [26]–[29]. HDAC inhibitors have also been tested to fight major human parasitic diseases such as leishmaniasis, malaria, schistosomiasis, toxoplasmosis, and trypanosomiasis (reviewed in [2] and [23]). Yet, these studies have highlighted the risk of cross-reactivity of the developed drugs with host (human) enzymes that can cause off-target effects.

To tackle this bottleneck, the use of structural data, obtained either from modeling or crystallographic/NMR studies, appears decisive [2]. Progress using this strategy has notably been made on *Plasmodium falciparum* HDAC1 (pfHDAC1), where chemical library screening and drug design studies, based on a homology model of this enzyme, have yielded inhibitors with anti-parasitic activity [30]–[33]. Yet, the modeling approach is not sufficient to account for all unique specificities in the active site that lead to the design of fully specific parasite inhibitors. This problem is particularly pronounced for many metazoan parasite epigenetic targets that, contrary to pfHDAC1, show strong sequence conservation with their human orthologs, notably for residues composing their active sites, reinforcing the need for detailed structural analysis.

To address this issue, we have characterized *Schistosoma mansoni* HDAC8 (smHDAC8) which has only a single active site amino acid substitution compared with human HDAC8 (hHDAC8). We have previously shown that *S. mansoni* encodes several HDACs, and treatment with generic HDAC inhibitors caused a global increase of protein acetylation in schistosomes and dose-dependent mortality of schistosomula and adult worms [34]–[36]. Importantly, all three *S. mansoni* class I HDACs (smHDAC1, 3 and 8) are expressed at all life-cycle stages, with *HDAC8* transcripts always being the most abundant [34], indicating that this latter enzyme is most likely a major target for the design of schistosome-specific inhibitors. The biological role of HDAC8 has long remained elusive. A recent study demonstrated however that human HDAC8 (hHDAC8) is involved in deacetylation of cohesin and mutations in hHDAC8 are linked with the Cornelia de Lange syndrome [37].

Here, we show that smHDAC8 is essential for parasite infectivity, reinforcing it as a potential epigenetic drug target. Crystal structures of smHDAC8 in native form and in complex with generic HDAC inhibitors reveal unexpected structural features in the smHDAC8 active site. Specifically, one conserved phenylalanine can adopt either a flipped-in or a flipped-out conformation. Strikingly, in human HDACs, only the flipped-in conformation is observed and appears to be fully constrained. Structure-based chemical screening further yielded a set of small-molecule inhibitors that, compared to generic HDAC inhibitors, showed decreased affinity for human HDACs, while retaining affinity towards smHDAC8. Notably, one of these inhibitors, J1075, not only forces the phenylalanine into the flipped-out conformation, but also induces apoptosis and mortality in schistosomes. Our data thus confirm the validity of the structure-based approach searching for new epigenome-targeting drug leads against schistosomiasis, but also provides a proof of concept for the treatment of other neglected diseases caused by eukaryotic pathogens.

## Results

### Down-regulation of *smHDAC8* expression in schistosomula causes a decrease in their capacity to survive and mature in infected mice

**Figure 1:**
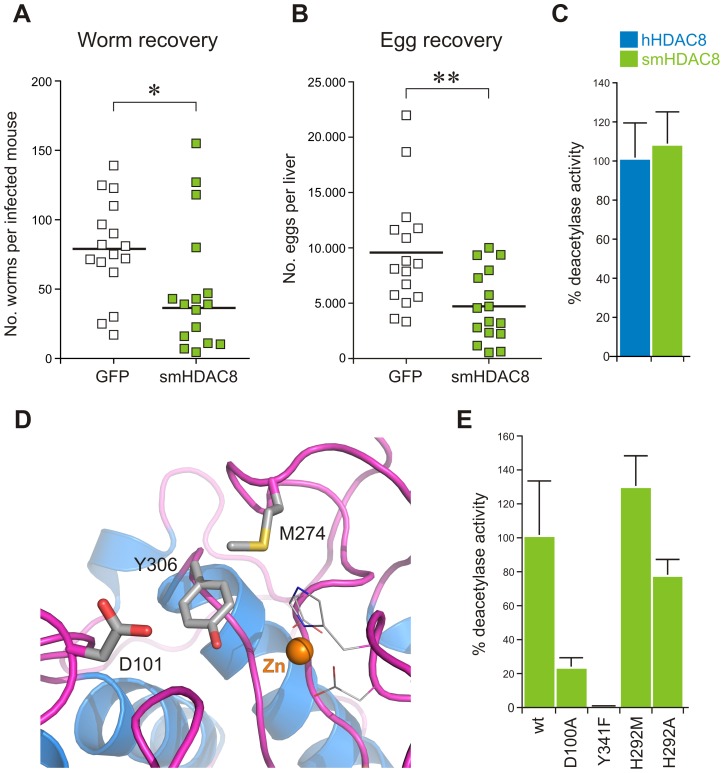
smHDAC8 is a functional acetyl-L-lysine deacetylase that is essential for parasite infectivity. (A) Down-regulation of *smHDAC8* decreases the number of adult worms recovered from infected mice. (B) The number of recovered eggs from the livers of infected mice is decreased. ‘*’, p<0.05; ‘**’, p<0.01. (C) Comparison of smHDAC8 and hHDAC8 deacetylase activities. Data indicate the average of relative deacetylase activity (hHDAC8 = 100%). Error bars represent the standard deviations (SD). (D) Close-up view of hHDAC8 active site. D101 and Y306, which participate in the hHDAC8 catalytic mechanism, and M274, which is replaced by a histidine in smHDAC8, are displayed. (E) Deacetalyse activities of smHDAC8 wild-type (wt), D100A, Y341F, and H292M mutants. Data indicate the average of relative deacetylase activity (smHDAC8 wt = 100%).

To investigate the importance of smHDAC8 in the schistosome life cycle, its expression was down-regulated by RNA interference. Transcript levels of smHDAC8 were reduced by 50% upon exposure of *in vitro* transformed schistosomula after incubation with dsRNA (“soaking” method) in culture, whereas schistosomula incubated with an irrelevant dsRNA encoding green fluorescent protein (GFP) showed no reduction in smHDAC8 transcripts. However, the microscopic examination of schistosomula treated with smHDAC8 dsRNA in culture for up to 7 days did not reveal any changes in tegument integrity, mortality, and motility (not shown). We therefore decided to infect mice (by i.v. injection) with schistosomula treated for 2 days in culture with dsRNA for smHDAC8, GFP or untreated. The mice were subjected to whole-body perfusion after 35 days to recover worms. Mouse livers and intestines were also recovered in order to determine tissue egg burdens. After 35 days post infection, mice infected with smHDAC8 knocked-down parasites showed a significant reduction in the number of recovered adult worms (an overall 50% reduction in comparison with the GFP dsRNA treated controls in three independent experiments) (Figure 1A). In addition, the tissue egg burden was reduced by 45%, again compared to the control (GFP) (Figure 1B). We conclude from these experiments that smHDAC8 is required for infection of the definitive host and plays a significant role in parasite homeostasis.

### smHDAC8 is a functional acetyl-L-lysine deacetylase

To characterize the role of smHDAC8, we first examined its enzymatic activity. For this purpose, the enzyme was overexpressed in *E*scherichia *coli* and purified through several chromatographic steps. Measurements of its deacetylase activity using a peptide with an acetylated L-lysine as a substrate showed that smHDAC8 exhibits a similar deacetylase activity to hHDAC8 (Figure 1C).

In human HDAC8, two residues have been shown to be critical for activity, D101 and Y306 [38], [39] (Figure 1D). The former residue is involved in binding the backbone of the incoming acetylated peptide substrate, whereas the hydroxyl group of the latter participates in the catalytic mechanism. Upon mutation of these two residues to alanine and phenylalanine, respectively, the corresponding mutated human HDAC8 showed a strong decrease in activity [38]. These two residues are fully conserved in smHDAC8 (D100 and Y341) and their respective mutation into alanine and phenylalanine also resulted in strong decrease/complete loss of deacetylase activity (Figure 1E).

An initial homology modeling of smHDAC8 based on the hHDAC8 structure suggested that there is only a single amino acid substitution in the catalytic pocket that distinguishes these two enzymes: the replacement of human M274 by a histidine (smHDAC8 H292). To investigate the importance of this substitution, we generated a “humanized” smHDAC8 protein by mutating H292 into methionine (H292M). In contrast to the previous mutants, this mutation did not affect smHDAC8 catalytic activity (Figures 1D and 1E). Mutation of this histidine to alanine (H292A) also yielded an active enzyme (Figure 1E), showing that this residue is not essential for activity. Nevertheless, the presence of a charged residue replacing a hydrophobic one in the active site of smHDAC8 suggested a key feature that could be exploited to design smHDAC8-specific inhibitors.

### Crystal structure of smHDAC8

To gain more precise structural knowledge of smHDAC8, we attempted crystallization of the native form of the enzyme. Crystals were obtained that belonged to space group P1 and which diffracted to 1.8 Å resolution (Table 1). The structure was solved by molecular replacement, using human HDAC8 as a search model. The initial model was further refined by several cycles of manual building and automatic refinement. The final model contains four independent non-crystallographic monomers (r.m.s.d. on Cα's from 0.1 to 0.6 Å; Figure S1A), and has good deviations from ideal geometry, with R-factor and R-free values of 0.15 and 0.19 (Table 1). Most of the residues could be built in density, except for a few disordered loops.

**Table 1:** Data collection and refinement statistics.

	Native smHDAC8	smHDAC8/SAHA	smHDAC8/M344	smHDAC8/J1038	smHDAC8/J1075
**Data collection**					
Space group	P1	P1	P1	P1	P1
Cell dimensions					
*a*, *b*, *c* (Å)	70.7, 70.8, 98.5	70.6, 70.6, 98.0	70.6, 70.6, 98.5	70.7, 70.8, 98.5	70.3, 70.3, 98.1
α, β, γ (°)	75.9, 78.3, 85.4	77.9, 75.5, 85.7	78.1, 75.4, 85.5	78.0, 75.5, 85.8	77.9, 75.6, 85.5
Resolution (Å)	1.8	2.0	1.65	2.2	2.0
*R*_sym_ or *R*_merge_	0.028 (0.127)*	0.087 (0.298)	0.058 (0.209)	0.072 (0.249)	0.124 (0.223)
*I*/σ*I*	26.5 (4.9)	21.1 (3.5)	29.8 (4.8)	15.7 (3.9)	17.2 (4.6)
Completeness (%)	97.2 (93.4)	97.5 (96.5)	96.9 (95.5)	93.3 (93.1)	97.1 (95.6)
Redundancy	1.9 (1.8)	2.9 (2.8)	3.3 (3.3)	1.9 (1.8)	2.7 (2.6)
**Refinement**					
Resolution (Å)	31.4–1.8	34.7–2.0	24.2–1.65	32.4–2.2	20.2–2.0
No. reflections	166,214	117,868	207,374	84,066	116,580
*R*_work_/*R*_free_	0.154/0.188	0.163/0.191	0.155/0.172	0.178/0.222	0.191/0.221
No. atoms					
Protein#	13,213	12,970	13,106	12,883	12,958
Ligand/ion	60	127	272	76	166
Water	1,532	926	1,171	551	632
*B*-factors					
Protein	22.8	28.0	23.3	25.0	24.3
Ligand/ion	27.4	37.4	38.9	50.5	42.7
Water	33.3	35.1	34.8	29.9	29.7
R.m.s. deviations					
Bond lengths (Å)	0.005	0.010	0.010	0.010	0.010
Bond angles (°)	0.881	0.940	0.940	0.970	0.950

* Values in parentheses are for highest-resolution shell.

# The number of protein atoms varies slightly for each structure, depending on the quality of the electron density for poorly folded regions.

**Figure 2:**
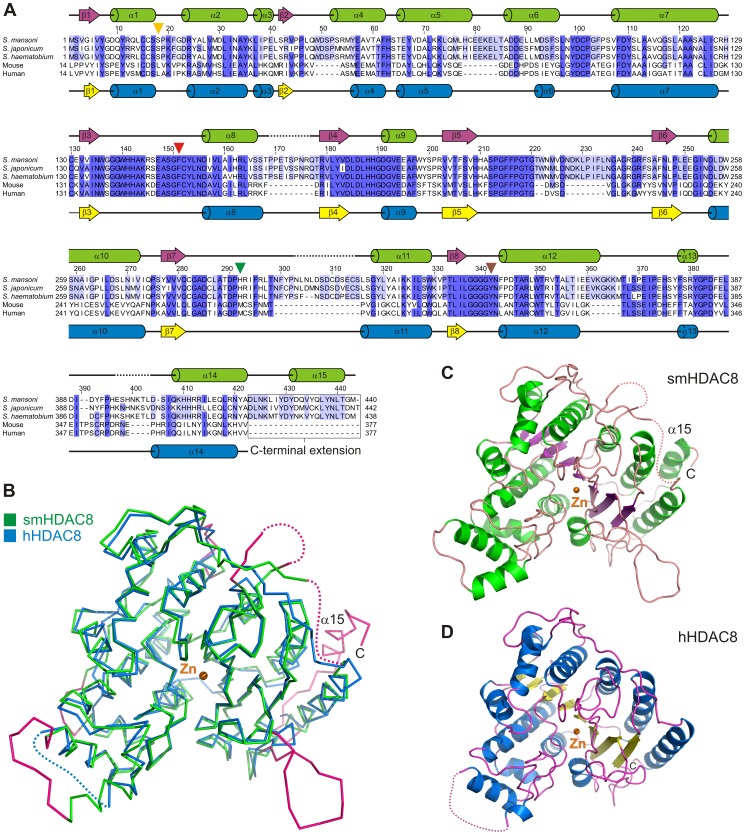
smHDAC8 adopts a canonical HDAC fold with specific external loops. (A) Structure-based sequence alignment of schistosome, mouse and human HDAC8 proteins. Sequences similarities are shown by levels of blue. Secondary structure elements found in smHDAC8 and hHDAC8 are shown above and below the alignment, respectively. Residues that could not be built in densities are depicted with a black dotted line. Important residues that participate in the specificity of the smHDAC8 active site are labeled with triangles. The numbering indicated above the alignment corresponds to smHDAC8. For clarity, the first thirteen residues of mouse and human HDAC8 have been removed. (B) Superposition of native smHDAC8 (green) and SAHA-inhibited hHDAC8 (blue; PDB 1T69) structures. Both enzymes adopt the same fold. smHDAC8 sequence insertions form specific external loops and C-terminus (colored in pink). The orange sphere represents the catalytic zinc ion (Zn). (C,D) Ribbon representations of smHDAC8 (C) and hHDAC8 (D) structures. Both enzymes adopt the same fold and their catalytic zinc ion is found at the same position.

The smHDAC8 adopts a fold similar to hHDAC8 [39] (r.m.s.d. on Cα's of 1.2 Å between the human and schistosome enzymes), forming a single α/β domain composed of a central, eight-stranded parallel β-sheet sandwiched by 15 α-helices (α1–α15) (Figures 2 and S1). The topology of the secondary structure elements is comparable to that observed in hHDAC8 with the exception of helix α15, which is a part of the C-terminal extension found in smHDAC8 (Figure 2).

Interestingly, HDAC8 enzymes from different *Schistosoma* species contain specific insertions compared to their mammalian orthologs (Figure 2A). Superposition of hHDAC8 and smHDAC8 structures reveals that these insertions extend external surface loops (Figure 2B). These extensions are located away from the active site, suggesting that they do not influence the catalytic mechanism directly and may form schistosome-specific protein/protein interaction surfaces. In addition, in human HDAC8 three ions are bound to the enzyme: the catalytic zinc at the bottom of the catalytic pocket, and two potassium ions. In smHDAC8 these three ions and their coordinating residues are conserved (Figure S1), thus reinforcing the similarity between the human and schistosome HDAC8 enzymes.

**Figure 3:**
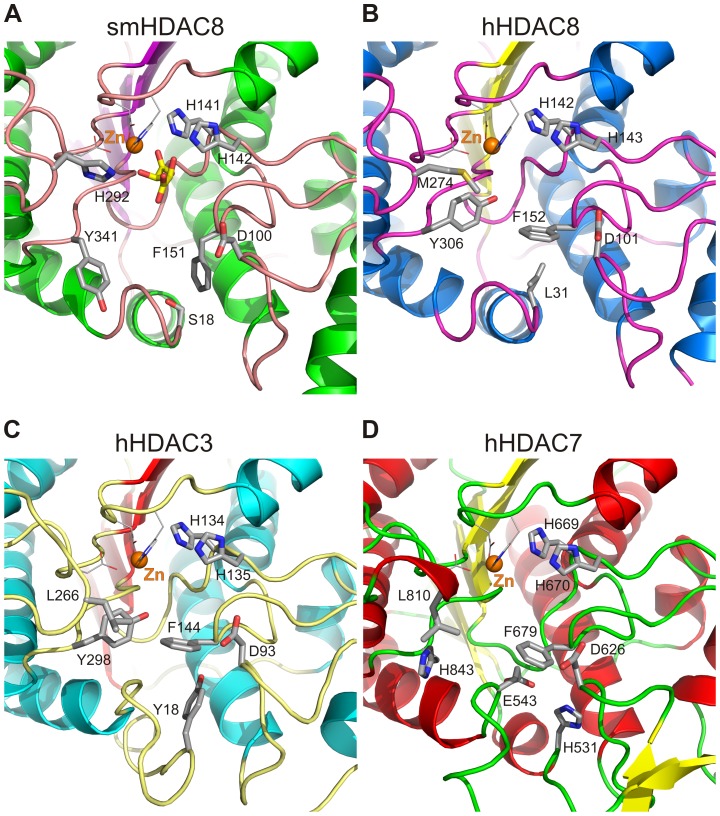
Flipping-out of smHDAC8 phenylalanine 151 (F151) cannot be accommodated by major human HDACs. Ribbon representation of the active sites of (A) smHDAC8, (B) hHDAC8 (PDB 1T67), (C) hHDAC3 (PDB 4A69), and (D) hHDAC7 (PDB 3COY). Residues participating in zinc binding, catalysis, and active site formation are shown as sticks. smHDAC8 F151 and its counterparts in human HDACs are shown as well as the residues that influence their conformation. Specifically, only the schistosome phenylalanine can adopt a favored flipped-out conformation. Of note, the active sites of hHDAC2 (PDB 3MAX) and hHDAC4 (PDB 2VQJ) have highly similar features as observed for hHDAC3 and hHDAC7, respectively.

Human HDAC8 has only been crystallized in the presence of small-molecule inhibitors or a peptidic substrate, suggesting that native hHDAC8 contains flexible parts, which may prevent crystallization. We were able to crystallize smHDAC8 in a non-inhibited form. However, we observed in our electronic density the unambiguous presence of an L-tartrate molecule provided by the crystallization buffer which was bound in the smHDAC8 active site where it coordinated the catalytic zinc ion (Figures 3A, S1 and S2). Crystals were also obtained in presence of the structurally related succinic acid, but these crystals showed poor diffraction, probably due to the absence of one coordinating hydroxyl group in this molecule compared to tartrate. Collectively, these results argue for an intrinsic flexibility of the HDAC8 active site which might be important for substrate recognition and/or catalytic activity.

### Specific architecture of the smHDAC8 catalytic pocket

Comparison of the active sites of hHDAC8 and smHDAC8 reveals several major changes between these proteins. First, as expected, we observe the replacement of M274 by H292 in smHDAC8 that diminishes the hydrophobic character of the pocket that normally accommodates the aliphatic part of the incoming acetylated lysine (Figures 3A and 3B). Further, while the position of smHDAC8 D100 is similar to that of its human counterpart, D101, we observe a change in conformation of the smHDAC8 Y341 side chain compared to hHDAC8 Y306. Specifically, the hydroxyl group of the latter is turned towards the zinc ion, where it interacts with the warhead of the inhibitors. In contrast, the smHDAC8 Y341 side chain points towards the rim of the catalytic pocket (Figures 3A and 3B).

The most striking and certainly least expected difference concerns the smHDAC8 F151 side chain that is turned away from the catalytic pocket and is inserted into a smaller hydrophobic pocket formed by loops that surround the active site (Figure 3A). In contrast to this flipped-out conformation of F151, the side chain of its human counterpart, hHDAC8 F152, is turned towards the active site adopting a flipped-in conformation in all inhibitor- and substrate-bound hHDAC8 structures deposited in the Protein Data Bank (PDB) (Figure 3B). Although this change could be due to the presence of inhibitors bound to hHDAC8, favoring a flipped-in conformation, careful inspection of hHDAC8 structures revealed that human F152 cannot adopt a flipped-out conformation. Indeed, this conformation is incompatible with the conformation of the L31 side chain that is itself locked in this conformation by its surrounding neighbors (Figure 3B). In the schistosome HDAC8 enzymes, this leucine is replaced by a serine (S18) (Figure 2A). This smaller residue enlarges the pocket accommodating the F151 side chain, enabling it to adopt its observed flipped-out conformation. This conformation of F151 appears favored over its flipped-in conformation since it contributes to strong Van der Waals contacts in the pocket where it is bound.

Interestingly, this phenylalanine is fully conserved in all human HDACs. Only the first HDAC domain of human HDAC6 contains a related tyrosine at this position (Figure S3). Strikingly, this phenylalanine is always found in a flipped-in conformation in all human HDACs structures solved so far (Figures 3B, 3C and 3D) [39]–[43]. Careful investigation of hHDAC2 and hHDAC3 structures, which are also class I HDACs, showed that the flipped-in conformation is also constrained due to the position of an invariant tyrosine provided by a specific loop of HDAC2 and HDAC3 (Y29 and Y18, respectively) (Figure 3C). In HDAC4 and HDAC7, class IIa HDAC family members, a flipped-out conformation of this phenylalanine would bring its side chain into a strongly unfavorable close vicinity (∼2.5 Å) of the side chains of a histidine and a glutamate residue (hHDAC7 H531 and E543) that also affect the hydrophobic character of the pocket used to accommodate the flipped-out conformation (Figure 3D).

Therefore, the flipped-out conformation of smHDAC8 F151 appears to be highly specific to the schistosome enzyme in contrast to human HDACs, suggesting that this feature, together with the specific replacement of human M274 by schistosome H292, could provide the basis for the design of specific inhibitors targeting smHDAC8. Specifically, the flipped-out conformation of F151 prevents this residue from participating in the formation of the narrow hydrophobic tunnel otherwise characteristic of hHDAC8 and which is required to accommodate the aliphatic part of the acetylated lysine substrate. The consequence of this change is a broader catalytic pocket in smHDAC8 that should be able to accommodate bulkier inhibitors as compared to hHDAC8.

### Ligand-induced gating of the catalytic pocket

**Figure 4:**
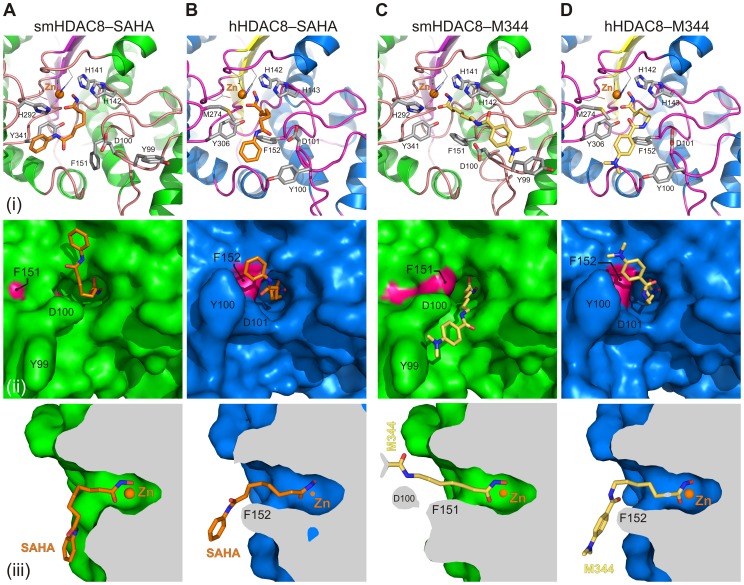
Ligand-triggered gating of the smHDAC8 active site pocket. Close-up view of the active sites of the (A) smHDAC8/SAHA, (B) hHDAC8/SAHA (PDB 1T69), (C) smHDAC8/M344, and (D) hHDAC8/M344 (PDB 1T67) complexes shown as ribbon and sticks (upper panels), surface view (middle panels), and side cut-away surface view (lower panels). The conformational changes of smHDAC8 Y99 and F151 compared to hHDAC8 Y100 and F152 strongly influence the binding modes of the SAHA and M344 inhibitors to these enzymes.

Towards this goal, we first determined the structures of smHDAC8 in complex with two generic HDAC inhibitors, SAHA (N-hydroxy-N′-phenyloctanediamide; [44]) and M344 (N-hydroxy-7-(4-dimethylaminobenzoyl)-aminoheptanamide; [45]), either by co-crystallization or by soaking. Crystallographic data at 2.0 and 1.65 Å, respectively, were collected. The refined models have low R-factors and show good deviations from ideal geometry (Table 1). SAHA and M344 are well-characterized HDAC inhibitors composed of a hydroxamate metal-binding warhead, a hydrophobic flexible linker domain, and a hydrophobic capping group (Figure S4 A and B). As expected, both inhibitors coordinate the catalytic zinc ion at the active site via their hydroxamate group. In addition, the hydroxamate head also interacts with H141, H142 and Y341 in a fashion similar to that observed in hHDAC8 (Figures 4 and S4). Specifically, Y341 now adopts the same conformation as seen for Y306 in inhibited human HDAC8 (Figure 4), a conformation that is most likely required not only for inhibitor binding, but also for substrate binding during catalysis as indicated by our mutation experiments (Figure 1E).

Importantly, upon SAHA binding, smHDAC8 F151 retains its flipped-out conformation, whereas, in human HDAC8, this phenylalanine contributes to the hydrophobic tunnel that accommodates the hydrophobic linker of the SAHA molecule (Figures 4A and 4B). The smHDAC8-bound SAHA adopts a kinked conformation that is different from the one observed for hHDAC8-bound SAHA. This different conformation in the case of the smHDAC8/SAHA complex is in fact enabled by the flipped-out conformation of F151. Indeed, a flipped-in conformation of this phenylalanine would clash with the hydrophobic linker of SAHA. This conformation further enables the hydrophobic capping group of SAHA to stack against the Y341 side chain (Figure 4A).

Strikingly, in the smHDAC8/M344 complex structure, we observe that F151 is changed from its flipped-out conformation to a flipped-in conformation, like that observed in hHDAC8 structures (Figure 4C). This flipped-in conformation of F151 contributes to M344 binding via Van der Waals contacts with the linker domain of this inhibitor. These findings clearly demonstrate that smHDAC8 F151 is able to adopt both flipped-out and flipped-in conformations. Curiously, despite the fact that F151 now adopts the same conformation as human F152, the M344 inhibitor adopts a different conformation when bound to each enzyme (Figures 4C and 4D): a straight conformation when bound to smHDAC8, and a kinked one when bound to hHDAC8, this latter conformation being highly similar to the one adopted by the hHDAC8-bound SAHA (Figures 4B and 4D). This difference is due, at least in part, to the specific conformation of smHDAC8 Y99, which is different from the conformation adopted by its human counterpart Y100 at the rim of the active site (Figures 4C and 4D). This change enables the capping group of smHDAC8-bound M344 to stack onto Y99, the conformation of the inhibitor being further stabilized through an interaction between its N10 atom and the smHDAC8 D100 carboxylate.

### Structure-guided design of specific inhibitors

We next used this structural information to perform a virtual screening to search for inhibitor scaffolds that would fit into the enlarged catalytic pocket of smHDAC8. These attempts, based on smHDAC8 active site specificities, aimed to identify novel chemical scaffolds that inhibit smHDAC8 activity. The large initial set of scaffolds identified by virtual screening was further analyzed by biochemical and biophysical assays (see below). Among the top-ranked inhibitors, we identified several linker-less aromatic hydroxamate derivatives (J1037, J1038, and J1075) that were subsequently used for structural characterization.

**Figure 5:**
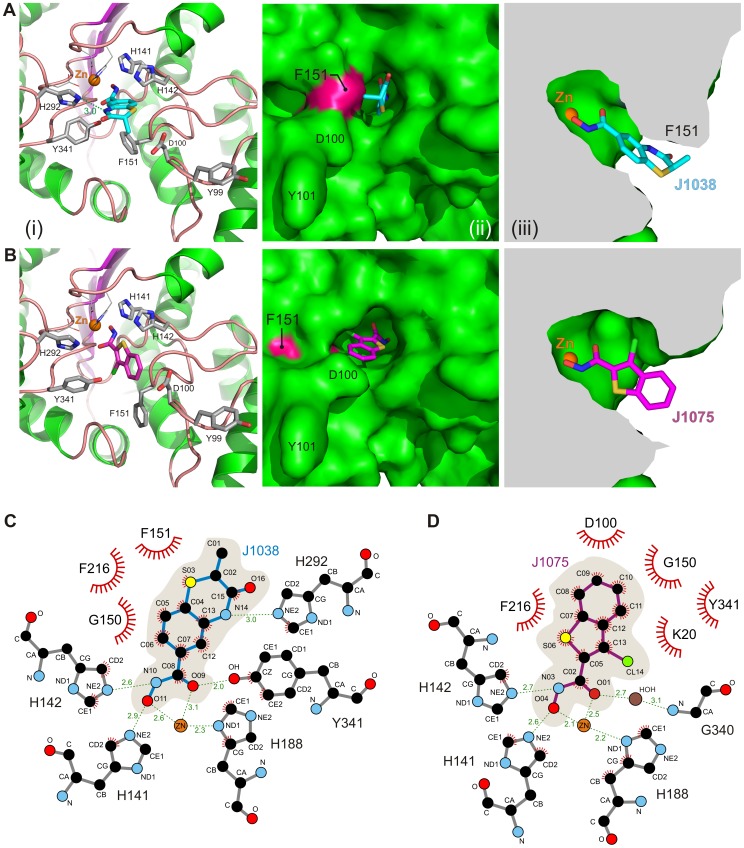
Binding modes of linker-less inhibitors J1038 and J1075 to smHDAC8. Structural mechanisms of smHDAC8 inhibitions by (A) J1038 and (B) J1075. The views are the same as those used in Figure 4. Both inhibitors make use of both common and specific interactions with smHDAC8. J1038 induces F151 flipping-in and interacts with H292, whereas J1075 constrains F151 in its flipped-out conformation but does not interact with H292. (C,D) Schematic views of the interactions formed by J1038 and J1075 with smHDAC8 active site zinc ion and residues.

Soaking of these inhibitors with the native form of smHDAC8 crystals enabled the collection of crystallographic data at high resolution for the J1038 (2-methyl-3-oxo-4H-1,4-benzothiazine-6-carbohydroxamic acid) and J1075 (3-chlorobenzothiophene-2-carbohydroxamic acid) inhibitors (2.2 and 2.0 Å resolution, respectively; Table 1). In contrast, J1037 caused an almost complete loss of the diffraction of the crystals. Inspection of the structures revealed that the J1038 and J1075 inhibitors bind in the active site of smHDAC8 and make use of the enzyme active site specificities. Although both inhibitors coordinate the catalytic zinc with their hydroxamate moieties in a fashion highly similar to SAHA and M344, their modes of binding to smHDAC8 otherwise use completely different features. Specifically, the annellated ring systems of both inhibitors adopt perpendicular conformations when bound to smHDAC8 (Figure 5).

Interestingly, in case of J1038, we observe that this inhibitor forms a hydrogen bond with the side chain of smHDAC8-specific H292, thus using one of the structural specificities of this enzyme (Figures 5A and 5C). However, binding of J1038, like M344, induces the F151 flipped-in conformation, this inhibitor being therefore unable to force this phenylalanine into its schistosome-specific conformation (Figure 5A).

In contrast, J1075 binding forces F151 to remain in its flipped-out conformation, but does not interact with H292 (Figures 5B and 5D). In addition, due to the presence of a bulkier chlorine atom in J1075, this inhibitor prevents Y341 from adopting its catalytic conformation and to coordinate the hydroxamate head (Figures 5B and 5D). In fact, in this structure Y341 adopts an intermediate conformation between the ones observed in the native and inhibitor-bound forms of smHDAC8.

**Figure 6:**
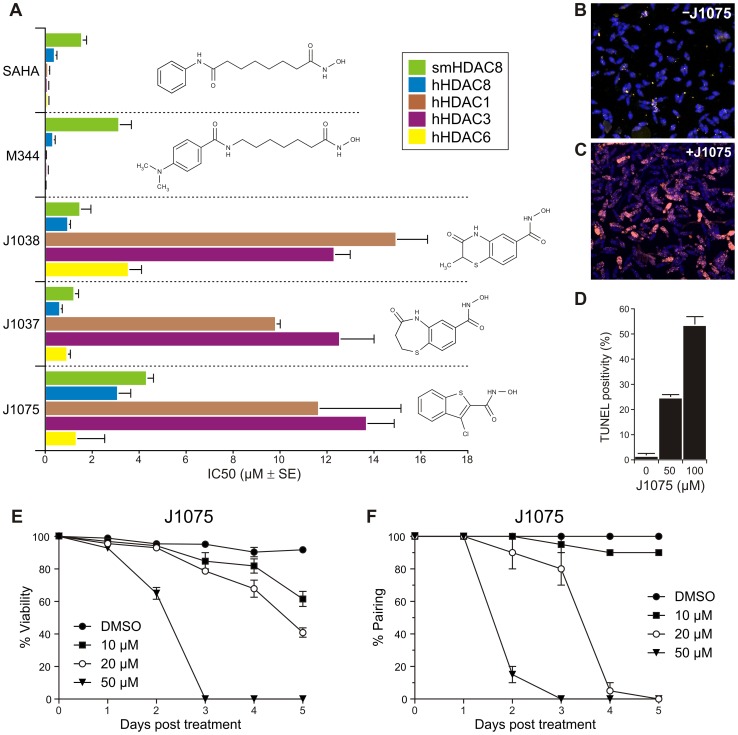
Designed small-molecule inhibitors show decreased specificity towards human HDACs and induce apoptosis in schistosomes. (A) IC_50_ values of SAHA, M344, J1038, J1037, and J1075 for smHDAC8 and human HDAC8, HDAC1, HDAC3, HDAC6 are plotted in graph. J1038 and J1075 show loss of specificities for human HDACs but not for smHDAC8. The results of three independent assays are shown, error bars represent the SD. (B,C) Merged TUNEL (pink) and DAPI (blue) staining of *S. mansoni* schistosomula incubated with DMSO alone (B) or with 100 µM J1075 dissolved in DMSO (C) for 96 h. (D) Quantification of TUNEL positivity of schistosomula incubated for 96 h with J1075 at 50 µM or 100 µM or with DMSO alone. The results of three independent assays are shown, error bars represent the SD. (E) Dose- and time-dependent mortality of schistosomula induced by J1075 inhibitor. *Schistosoma mansoni* schistosomula (1000 per well) were incubated in 1 mL of culture medium with varying quantities of J1075 inhibitor or the solvent (DMSO). The results of three independent assays are shown; error bars represent the SD. (F) J1075-triggered separation of *S. mansoni* adult worm pairs in culture. The paired status of male and female adult worms was assessed daily in the presence of varying quantities of J1075 or the solvent (DMSO). The results of three independent assays are shown, error bars represent the SD.

Thus, although J1038 and J1075 similarly bind the catalytic zinc ion of smHDAC8 through their hydroxamate moieties, they otherwise build their binding specificity on completely different features. When these inhibitors, as well as J1037, were investigated using differential scanning fluorimetry, we observed that these molecules were able to stabilize smHDAC8 to the same extent as SAHA and M344 (Figure S5). Importantly, these novel inhibitors showed IC_50_ values for smHDAC8 in the same range as for SAHA and M344 (Figure 6A). For human HDAC8, IC_50_ values were about two-fold higher than those obtained with SAHA and M344. Strikingly, the largest changes in IC_50_ values with these new inhibitors were observed with human class I HDAC1 and HDAC3 but also class II HDAC6, with increased IC_50_ values up to 100 fold (Figure 6A).

### Linker-less hydroxamate derivative, J1075, causes apoptosis and death of schistosomes

We previously showed [36] that pan-HDAC inhibitors such as trichostatin A or valproic acid could induce the death of schistosomula or adult worms in culture and that worm death was due to the induction of apoptosis as evidenced by TUNEL staining and the activation of effector caspase activities. In order to determine whether the HDAC8-selective inhibitors J1037, J1038 and J1075 would be able to have similar effects, schistosomula larvae were exposed to these inhibitors in culture for up to 96 h followed by TUNEL staining to detect the induction of cell death. These inhibitors had completely different effects: whereas J1037 and J1038 did not appear to affect the parasites up to 100 µM, these experiments revealed TUNEL staining in schistosome cells and parasite death upon J1075 exposure at 50 and 100 µM after 96 h (Figures 6B, 6C and 6D). In comparison, the pan-HDAC inhibitor SAHA induces a positive TUNEL reaction within 48 h at the same concentrations (Figure S6), as does M344. We initially hypothesized that the faster effect observed with SAHA and M344 was due to their targeting of multiple HDACs in schistosomes. However, we also noticed that the commercial J1075 sample we used (supplied by Enamine, Kiev, Ukraine) contained many impurities, and we decided to synthesize this inhibitor *de novo*. Strikingly, the pure, synthesized inhibitor proved much more effective: when incubated with schistosomula, it induced 100% mortality at 50 µM within 3 days and was active at 10 µM (Figure 6E). Moreover, when incubated with adult worm pairs, it induced separation of the male and female worms, causing complete separation of worm pairs within 3 days at 50 µM and 5 days at 20 µM (Figure 6F). Together, these results show that a selective inhibitor of smHDAC8 has similar toxic effects on schistosomes like pan-HDAC inhibitors, reinforcing the interest of this enzyme as a therapeutic target and of J1075 as a lead compound for drug development.

## Discussion

The current success observed in targeting the human epigenome for the development of anti-cancer drugs has broad implications, notably concerning the discovery of novel drugs to cure human parasitic diseases that cause millions of deaths yearly. However, the risk of cross-reactivities of parasite-targeting drugs with human enzymes requires careful design of inhibitors. Comparative structural analyses were expected to be essential for this kind of approach. Our results demonstrate that such an approach is valid and highly promising.

We have applied our strategy to the HDAC8 enzyme from *Schistosoma mansoni*. We have shown previously that *S. mansoni HDAC8* is expressed at all life stages of schistosomes. Our current results, which indicate a marked reduction in the number of recovered adult worms after infection of mice with schistosomula knocked-down for *smHDAC8*, confirm the importance of this protein as a therapeutic target. However, sequence alignment showed almost complete conservation of active site residues between schistosome and human HDAC8.

By comparing the structure of *S. mansoni* HDAC8 in native and inhibited forms with the structures of inhibited human HDAC8, we observe, despite this high sequence conservation of active site residues, different changes in the active site that increase the possibility of designing specific inhibitors. Notably, two substitutions, M274→H292 and L31→S18, directly and indirectly modify the shape and physico-chemical properties of the smHDAC8 active site (Figures 3 and 4).

**Figure 7:**
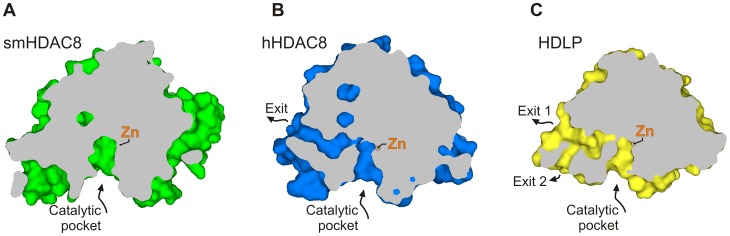
smHDAC8 lacks a putative acetate exit channel. The cut-away surface views of (A) smHDAC8, (B) hHDAC8, and (C) bacterial HDAC-like HDLP. These snapshots reveal the absence of typical, continuous internal cavity connecting the active site with the outside environment in smHDAC8. Careful inspection of the smHDAC8 structure did not reveal an obvious alternative channel at another location. The catalytic zinc ion at the active site is depicted by an orange sphere.

The consequence of the S18 substitution concerns F151 that adopts a flipped-out conformation that cannot be accommodated in human HDAC8 and other major human HDACs. The reason for this preferred flipped-out conformation is unclear. It has been suggested that human HDAC8 [39] contains an exit channel for the cleaved acetate molecule that starts at the bottom of the catalytic cavity and goes through the enzyme, a feature also observed in bacterial histone deacetylase-like proteins (HDLPs) [46], [47]. Surprisingly, this internal channel is absent in smHDAC8 because of several substitutions introducing residues with bulkier side chains (Figure 7). Therefore, the flipping-out of F151 could provide an alternative to the exit channel and facilitate exit of the acetate product. We cannot exclude, alternatively, that the F151 flipping-out enables the recognition of a structurally different substrate. Such plasticity could be used to adapt to various epigenetic regulations that drive the intricate parasitic life cycle that presents several phenotypically very different stages.

An important result from our study is that the novel linker-less inhibitors designed according to our initial structures are able to make use of the smHDAC8 active site pocket specificities, retaining an inhibitory effect on smHDAC8, but showing significant loss in potency for major human HDACs such as HDAC1, HDAC3, and HDAC6 as off-targets. Although IC_50_ values also increased for human HDAC8, this phenomenon was less pronounced than for the other human HDACs tested. Our structural data suggest that HDAC8 enzymes display some flexibility at their active sites, which could explain that small molecules (e.g. L-tartrate in our native form and inhibitors in the other cases) coordinating the catalytic zinc are required to stabilize the protein and support crystallization. This intrinsic flexibility could contribute to the better accommodation of our linker-less inhibitors by hHDAC8 than other human HDACs. However, our structures of smHDAC8 bound to these novel inhibitors reveal that this enzyme could accommodate even bulkier linker-less inhibitors. Our current inhibitors therefore form the building blocks for the design of second generation inhibitors that should show increased specificity towards smHDAC8 versus hHDAC8.

Of note, whereas the increase of IC_50_ values observed with J1075 for human HDACs is certainly due to the inability of these HDACs to have a phenylalanine in the flipped-out conformation, the reason for this increase with J1038, and possibly for the related J1037 inhibitor, is most likely different. In smHDAC8 we have seen that H292, which interacts with J1038 through a hydrogen bond, is replaced by a methionine in hHDAC8, which is certainly not favorable for J1038 binding. In human HDAC2 and HDAC3 (but also in human HDAC4 and HDAC7), a leucine residue, protruding deeper into the active site pocket, is found at the position of M274 that should cause even stronger hindrance for the optimal binding of J1038, as suggested by our inhibition experiments. Since HDAC6 also shows increased IC_50_ values, it is likely that a similar mechanism is at play with this enzyme. However, the fact that HDAC6 structure is unknown and that this protein is composed of two HDAC domains arranged in tandem currently impairs its accurate modeling and prevents an interpretation of our results in molecular terms.

Our complementary *in vivo* studies support our structural and biochemical results, J1075 being able to induce apoptosis and death of schistosomes, as well as the unpairing of adult worms. Since the maintenance of adult worm pairs is necessary for egg-laying, which in turn is responsible for the pathological effects of schistosome infection, worm unpairing may indicate that the inhibition of smHDAC8 will lead to reduced morbidity. The reason for the apparent inefficiency of J1037 and J1038 in both assays remains unclear. Poor uptake or metabolic inactivation may be possible explanations for these observations. The design of more specific inhibitors based on our current structural data should help to reduce the possible residual toxicity in human cells and increase the capacity to kill schistosomes.

Most HDAC inhibitors that are currently approved for use in humans, or are in clinical trials are, at best, moderately selective for an HDAC class [48] and not for one particular enzyme. Our objective is to maximize the selectivity of the inhibitors we develop in order to avoid possible side-effects that can be associated with treatment with HDAC inhibitors. For instance, side-effects of treatment with Vorinostat (SAHA) include fatigue, nausea and diarrhea, but the most severe adverse effect observed with HDAC inhibitors has been cardiac toxicity, including ventricular arrhythmia (see [49] for review). However, the dosing schedule for HDAC inhibitors in cancer therapy usually involves multiple doses given over a long period. The objective for the treatment of schistosomiasis is to develop a single dose strategy, as is the case for praziquantel. Together with selectivity for the schistosome enzyme over human HDACs, this should ensure that the side effects of treatment are minimal.

So far, our study has been dedicated to the inhibition of *S. mansoni* HDAC8. However, this study is expected to have broader implications. Notably, smHDAC8 S18, which enables F151 flipping out, is conserved in all schistosome HDAC8 enzymes sequenced so far, including *S. japonicum* and *S. haematobium* (Figure 2A), as well as another trematode species, *Clonorchis sinensis* (Figure S7). The same is true for smHDAC8 H292. Homology modeling of the HDAC8 structures from these different species based on sequence alignment and our structural data did not suggest any amino acid substitution in these enzymes that could prevent, directly or indirectly, F151 to adopt its flipped-out conformation. These observations are of paramount importance since they imply that smHDAC8-specific inhibitors should also inhibit the corresponding enzymes of *S. japonicum*, *S. haematobium* and *C. sinensis*, and possibly those of other trematode species that affect different populations worldwide. Moreover, *S. haematobium* and *C. sinensis* are also linked with the progression of cancer (reviewed in [50]). Hence, development of drugs fighting these infections should also have an implication in cancer prevention.

Strikingly, smHDAC8 S18 but also smHDAC8 H292 are conserved in the recently published HDAC8 sequences of the cestodes *Echinococcus multilocularis*, *Echinococcus granulosus* and *Taenia solium* [51] that also cause serious human diseases (Figure S7). Modeling of the structures of HDAC8 from *E. multilocularis*, *E. granulosus* and *T. solium* reveals that the active sites of these enzymes are highly conserved compared to that of smHDAC8. Specifically, as for the various schistosome species, the flipped out conformation of the phenylalanine corresponding to smHDAC8 F151 should also be conserved in these enzymes. In addition, the position of the histidine corresponding to smHDAC8 H292 should be the same as in the schistosome enzyme. These results suggest that the specific drugs developed against smHDAC8 could in fact be used in a pan-platyhelminth treatment strategy.

Taken together, our results provide the molecular basis for specific targeting a parasitic epigenetic enzyme to cure parasitic diseases by combining high resolution structural data with biochemical and *in vivo* studies. These results pave the way for similar studies on various platyhelminth epigenetic players, but also on other major parasites that, among others, cause malaria, leishmaniasis, Chagas disease, toxoplasmosis, and trypanosomiasis.

## Materials and Methods

### Ethics statement

Experiments involving mice were carried out according to the European directive 2010/63/EU concerning the use of animals for scientific purposes. Specifically, Brazilian national guidelines set out in the Law 11794/2008 were followed, stipulating the conditions for the use of animals in scientific research and setting up the National Council for the Control of Animal Experimentation (CONCEA) requiring the establishment of ethics committees on the use of animals (CEUA) by institutions under operational standards set out in Decree 6899/2009,2. Accordingly, animal experiments carried out in this work were approved by the Ethics Review Committee for Animal Experimentation (CETEA) of Universidade Federal de Minas Gerais number 185/2006.

### RNAi-mediated knockdown of *smHDAC8*

The smHDAC8 specific PCR primers containing the T7 promoter-tail amplified a ∼500 bp fragment (dsRNA-smHDAC8 Forward 5′-TAATACGACTCACTATAGGGGATACGCCTTGGTCAT GGAT-3′, and dsRNA-smHDAC8 Reverse 5′-TAATACGACTCACTATAGGGTGTACCG GGAAAGAAACCAG-3′). A GFP nonspecific ∼500 bp control was used (dsRNA-GFP Forward 5′-TAATACGACTCACTATAGGGTCTTCAAGTCCGCCATG-3′ and dsRNA-GFP Reverse 5′-TAATACGACTCACTATAGGGTGCTCAGGTAGTGGTTGTC-3′). Double-stranded RNAs (dsRNAs) were synthesized *in vitro* from schistosomula cDNA using T7 RiboMAX Express RNAi Kit (Promega). Schistosomula were obtained by mechanical transformation of cercariae [52] of the LE strain of *S. mansoni* and cultured in supplemented MEM medium. Schistosomula (2000 per assay point) were exposed to 100 µM of dsRNA and cultured for 2 days at 37°C under CO_2_. For qRT-PCR (minimum of 3 experiments) total RNA was extracted using the RNeasy Mini Kit (Qiagen). Total RNA (100 ng) was used to synthesize cDNA with Superscript III cDNA Synthesis kit (Life Technologies). *S. mansoni* cytochrome oxidase was used as an endogenous normalization control in all tested samples (GenBank AF216698) (COX Forward 5′-TACGGTTGGTGGTGTCACAG-3′ and COX Reverse 5′-ACGGCCATCACCATACTAGC-3′). Quantitative RT-PCR was carried out using Power SybrGreen PCR Master Mix (Life Technologies) in an AB7900 Real Time PCR System (Life Technologies). Negative controls used were GFP and two internal controls assessing both possible genomic DNA contaminations (no reverse transcriptase) and purity of the reagents (no cDNA). Each experiment was repeated 3–5 times and analyzed using the ΔΔCt method [53].

### *In vivo* experiments – Recovery of adult worms and eggs

SWISS Webster mice were infected with 300 schistosomula treated with dsRNA for 2 days (3 independent experiments, 5 or 6 animals per group). After 35 days parasites were perfused according to standard protocols [54]. Livers from infected animals were weighed and eggs counted after digestion with KOH.

### Cloning, protein expression and purification

The full-length cDNA for smHDAC8 [34] was PCR-amplified using the forward primer smHDAC8-N (5′-GGATATCCATATGTCTGTTGGGATCGTTTATG-3′) and the reverse primer smHDC8-CNS (5′-CGCGGATCCCATACCAGTTAAATTATATAATTG-3′). The amplified gene was cloned between the NdeI and BamHI sites of the pnEA/tH vector encoding a C-terminal thrombin cleavage site followed by a His-tag [55]. The mutants were generated using standard nested protocols and inserted into the same vector.

Overexpression was carried out in *E. coli*BL21(DE3) cells in Terrific Broth (TB) medium. Induction was done at 37°C by adding 0.5 mM final isopropyl-1-thio-β-D-galactopyranoside (IPTG, Euromedex), in presence of 100 µM ZnCl_2_. Harvested bacteria were resuspended in lysis buffer (50 mM KCl, 10 mM Tris-HCl pH 8.0) and lysed under high pressure (18000 psi) in a Microfluidizer Processor M-110EH (Microfluidics). The lysate was clarified by ultracentrifugation (40000 rpm, Ti45 Beckman) for 1 h. The supernatant was loaded onto Talon Metal affinity resin (Clontech) pre-equilibrated with the lysis buffer. The His-tagged smHDAC8 protein was released from the Talon resin by thrombin treatment and subsequently loaded onto a 1-mL HiTrap Q FF (GE Healthcare) column pre-equilibrated with the lysis buffer. The protein was eluted by a linear gradient of KCl (50 mM to 1 M KCl) and then loaded onto HiLoad 16/60 Superdex 200 gel filtration column (Amersham Bioscience) equilibrated in 50 mM KCl, 10 mM Tris-HCl pH 8.0, and 2 mM DTT. The protein was concentrated with an Amicon Ultra centrifugal filter units (Millipore) to reach a final concentration of 2 mg/ml as assayed by the Bio-Rad Protein Assay reagent (Bio-Rad).

### Crystallization and data collection

Crystallization trials were performed using both sitting and hanging drop vapor diffusion techniques. Diffraction-quality crystals were obtained at 17°C after three to four days by mixing equal volumes of smHDAC8 with reservoir solution composed of 21% PEG 3350 (Fluka) and 0.2 M Na^+^/K^+^ L-tartrate. The smHDAC8/SAHA complex was formed by incubating the smHDAC8 protein (2 mg/ml) with SAHA (5 mM resuspended in DMSO) at 4°C for 1 h. Crystals were grown in the same conditions as described for native smHDAC8.

Crystals of the complexes of smHDAC8 with the inhibitors M344, J1038 and J1075 inhibitors were produced by soaking native smHDAC8 crystals in mother liquor supplemented with the corresponding inhibitor (10 mM resuspended in DMF) for 20 hours. Crystals used for X-ray data collection were briefly transferred in reservoir solution supplemented with 20% glycerol and flash-frozen in liquid nitrogen. All data obtained in this project were collected at 100 K on ESRF beamline ID23-1 and SOLEIL beamline PROXIMA1.

### Structure determination, model building and refinement

All data were processed and scaled using HKL2000 [56]. The structure of native smHDAC8 at 1.8 Å resolution was solved by molecular replacement with Phenix [57] using the hHDAC8 structure (PDB 1T69) as a search model. The crystals of the smHDAC8/SAHA, smHDAC8/M344, smHDAC8/J1038, and smHDAC8/J1075 complexes all belonged to the same space group and had the same unit cell as native SmHDAC8 crystals, and only rigid-body refinement was used to adapt to the slight differences in unit cell constants.

The initial models were refined through several cycles of manual building using COOT [58], [59] and automated refinement with Phenix [57] and Buster [60]. All models have low R and R-free factors, good deviations from ideal geometry (Table 1), and no Ramachandran outliers. Crystal structures solved in this project have been deposited under the codes 4bz5, 4bz6, 4bz7, 4bz8 and 4bz9 in the Protein Data Bank. Alignments were generated with JalView [61], structural panels in figures were generated with PyMOL (DeLano Scientific), and schematic interactions of the inhibitors with smHDAC8 were generated with LigPlot [62]).

### Virtual screening

Virtual screening was carried out using the native smHDAC8 structure as template. We screened the Enamine purchasable compound library (1.587.660 compounds) for potential smHDAC8 inhibitors by a stepwise virtual screening procedure. First we screened for compounds containing hydroxamic acid groups or derivatives which are known as zinc binding motifs. The molecular weight was restricted to 300 Daltons to identify smaller lead-like compounds. In total 25 molecules were retrieved, their 3D structures were generated within MOE 2008.10 (Chemical Computing Group, Montreal, Canada) and docked into the smHDAC8 structure using GOLD4.1 docking program [63]. ChemScore was used as scoring function due to the success of ChemScore in earlier HDAC dockings [64]. The docking region was defined within a radius of 15 Å around the zinc ion. The docking showed that the top-ranked hydroxamates are well coordinated to the zinc ion. The top-ranked compounds (J1037, Enamine compound ID Z285139392; J1038, Enamine compound ID Z253047366; J1075, Enamine compound ID Z1269129447) were further considered for inhibition assays *in vitro* and for structural work.

### J1075 synthesis

The hydroxamic acid (J1075) was synthesized through a fast two-step synthesis, starting from cinnamic acid which is transformed into 3-chlorobenzo[b]thiophene-2-carbonyl chloride by a ring closure using thionyl chloride as solvent/reagent, and pyridine as a catalyst (by a modification of the procedure described [65]. The carbonyl chloride was further reacted into the hydroxamic acid 3-chloro-N-hydroxybenzo[b]thiophene-2-carboxamide (J1075) using a procedure similar to one already described in [66].

### Activity and inhibition assays

Human and smHDAC8 activity testing was carried out with the HDAC8 Fluorimetric Drug Discovery Kit (Fluor de Lys(R)-HDAC8, BML-KI178) from Enzo Life Sciences according to the manufacturer's instructions with a substrate concentration of 50 µM. Fluorescence was measured in a plate reader (BMG Polarstar) with excitation at λ = 390 nm and emission at λ = 460 nm. Inhibition data for human HDACs 1, 3 and 6 were obtained according to published procedures with Z(Ac)Lys-AMC as the substrate and trypsin as the developing agent [67]. HDAC1, 3 and 6 were purchased from BPS Bioscience.

### Differential scanning fluorimetry assays

The full-length smHDAC8 protein was added to a final concentration of 0.25 mg/ml in the incubation buffer (50 mM KCl, 10 mM Tris-HCl pH 8.0, and 5× SYPRO Orange (Invitrogen) fluorescent dye). The final volume was 30 µl/well in a PCR plate. The temperature gradient was performed in the range of 20–95°C with a heating rate of 0.5°C/min, using a MiniOpticon real-time detection system (Bio-Rad) with excitation at 490 nm and emission at 530 nm. The midpoint temperature values of the unfolding transition (Tm) were generated with help of Opticon Monitor 3 (Bio-Rad) program. The ΔT_m_ of the smHDAC8 protein for a specific inhibitor was calculated as the difference between the Tm values of the inhibitor-bound and inhibitor-free proteins. All the assays were done in triplicate.

### Treatment of schistosomula with HDAC inhibitors in culture

A Puerto-Rican strain of *S. mansoni* was maintained in *Biomphalaria glabrata* snails and golden hamsters (*Mesocricetus auratus*). Cercariae released from infected snails were harvested on ice, washed 3 times by resuspension in 30 ml of Hank's Balanced Salt Solution (Invitrogen) in a corex tube (Corning) and centrifugation for 10 min. at 1500 g. Schistosomula were obtained *in vitro* [52] and were maintained in culture for up to 8 days under the conditions previously described [36]. Detection of DNA strand breaks in TSA-treated schistosomula was done using the dUTP nick end labelling (TUNEL) method using the *In Situ* Cell Death Detection Kit TMR Red (Roche). The method designed for cell suspensions was followed as described in the manufacturer's instructions with the modifications described previously [36]. Positively labelled schistosomula were counted manually under fluorescence microscopy. Alternatively, the viability of schistosomula was visually estimated on a daily basis using an optical microscope (Leica DMIL) following the distinct morphological differences between dead and viable schistosomula. Loss of mobility, obvious tegumental deformation and a granular appearance were the main criteria. For each condition, an aliquot of approximately 150 larvae was observed and the non-viable larvae were counted. Three counts were independently performed for each experiment. Adult worm pairing was assessed visually for 8 worm pairs maintained in culture under the same conditions as for schistosomula. For the viability and pairing assays three independent experiments were carried out in duplicate.

## Supporting Information

Figure S1 Structural features of smHDAC8. (A) Four non-crystallographic smHDAC8 monomers (A to D) are observed in the asymmetric unit. Root-mean-square deviation (r.m.s.d.) between each monomer range from 0.1 to 0.6 Å. (B) Ribbon representations of the smHDAC8 structure. The L-tartrate molecule bound in the active site is drawn with sticks. Orange sphere, catalytic zinc ion; blue spheres, potassium ions. (C) Close-up view of both the smHDAC8 active site and potassium-binding site A (K_A_ site). Residues involved in zinc and K_A_ binding are drawn in stick representation. The carbon atoms of proteins side chains are in grey, whereas the carbon atoms of L-tartrate are in yellow. The associated multiple alignment shows the conservation of the residues coordinating the zinc ion (orange circles) and the K_A_ ion (blue circles) among HDAC8 family members as well as the HDAC-like bacterial protein HDLP. Note that the tartrate molecule also coordinates the zinc. (D) Close-up view of the potassium binding site B (K_B_ site). Residues involved in the K_B_ ion coordination are drawn in sticks. The red sphere represents a water molecule. The associated alignment shows conservation of residues coordinating the K_B_ ion (blue squares).
(TIF)

Figure S2 Interaction between native smHDAC8 and the L-tartrate molecule. (A) Chemical structure of L-tartrate molecule. (B) Schematic view of the interactions made by the tartrate molecule as well as the smHDAC8 catalytic zinc ion. The hydrogen bonds and salt bridges observed in the crystal structure are shown with green dotted lines together with their associated distances (Å). Black circles, carbon atoms; red circles, oxygen atoms; blue circles, nitrogen atoms; orange circle, zinc atom. Proteins side chains are in grey, while the L-tartrate scaffold is in yellow.
(TIF)

Figure S3 Sequence alignment of smHDAC8 and all human HDACs in the vicinity of the phenylalanine (indicated by a triangle) which adopts either a flipped-in or a flipped-out conformation. Note that hHDAC6 contains two deacetylase domains, which are here designed as hHDAC6-I and hHDAC6-II. The phenylalanine is conserved in all human HDACs, except in the first HDAC domain of hHDAC6 where it is replaced by a tyrosine. Sequence conservation is indicated with blue levels.
(TIF)

Figure S4 Molecular interactions between smHDAC8 and the generic hydroxamate inhibitors SAHA and M344. (A,B) Chemical structures of SAHA and M344. (C,D) Schematic view of the interactions made by the SAHA and M344 inhibitors with smHDAC8. The same color codes are used than in Figure S2.
(TIF)

Figure S5 Thermal stabilization of smHDAC8 in presence of the SAHA, M344, J1038, J1037, and J1075 inhibitors. The values represent the mean difference of melting temperatures (ΔTm) observed for smHDAC8 in presence and absence of inhibitors plotted and calculated from three independent assays. Error bars represent the standard deviation (SD).
(TIF)

Figure S6 Quantification of TUNEL positivity of schistosomula incubated for 48 h with 0, 20, 50 or 100 µM of SAHA dissolved in DMSO. Results are expressed as the mean values of two independent experiments, with error bars giving the SD.
(TIF)

Figure S7 Sequence alignment of schistosome, *Echinococcus multilocularis*, *Echinococcus granulosus*, *Taenia solium*, *Clonorchis sinensis* and human HDAC8 proteins. Sequences similarities are shown by levels of blue. Important residues that participate in the specificity of *Schistosoma mansoni* HDAC8 active site and are conserved in the other platyhelminthes HDAC8 proteins are labeled with triangles. The numbering indicated above the alignment corresponds to smHDAC8.
(TIF)

## References

[1] Hotez PJ, Pecoul B (2010) “Manifesto” for advancing the control and elimination of neglected tropical diseases. PLoS Negl Trop Dis 4: e718. 20520793 10.1371/journal.pntd.0000718 PMC2876053

[2] Andrews KT, Haque A, Jones MK (2012) HDAC inhibitors in parasitic diseases. Immunol Cell Biol 90: 66–77. 22124373 10.1038/icb.2011.97

[3] Morens DM, Folkers GK, Fauci AS (2004) The challenge of emerging and re-emerging infectious diseases. Nature 430: 242–249. 15241422 10.1038/nature02759 PMC7094993

[4] Prasad KJ (2010) Emerging and re-emerging parasitic diseases. J Int Med Sci Acad 23: 45–50.

[5] King CH (2010) Parasites and poverty: The case of schistosomiasis. Acta Trop 113: 95–104. 19962954 10.1016/j.actatropica.2009.11.012 PMC2812649

[6] Brown M (2011) Schistosomiasis. Clin Med 11: 479–482. 10.7861/clinmedicine.11-5-479 PMC4954246 22034712

[7] Ross AG, Bartley PB, Sleigh AC, Olds GR, Li Y, et al (2002) Schistosomiasis. New Engl J Med 346: 1212–1220. 11961151 10.1056/NEJMra012396

[8] Gray DJ, Ross AG, Li Y-S, McManus DP (2011) Diagnosis and management of schistosomiasis. BMJ 342: d2651. 21586478 10.1136/bmj.d2651 PMC3230106

[9] Dömling A, Khoury K (2010) Praziquantel and Schistosomiasis. ChemMedChem 5: 1420–1434. 20677314 10.1002/cmdc.201000202

[10] Ismail M, Botros S, Metwally A, William S, Farghally A, et al (1999) Resistance to praziquantel: direct evidence from *Schistosoma mansoni* isolated from Egyptian villagers. Am J Trop Med Hyg 60: 932–935. 10403323 10.4269/ajtmh.1999.60.932

[11] Doenhoff MJ, Kusel JR, Coles GC, Cioli D (2002) Resistance of Schistosoma mansoni to praziquantel: is there a problem? Trans Roy Soc Trop Med Hyg 96: 465–469. 12474468 10.1016/s0035-9203(02)90405-0

[12] Doenhoff MJ, Cioli D, Utzinger J (2008) Praziquantel: mechanisms of action, resistance and new derivatives for schistosomiasis. Curr Opin Infect Dis 21: 659–667. 18978535 10.1097/QCO.0b013e328318978f

[13] Norton AJ, Gower CM, Lamberton PH, Webster BL, Lwambo NJ, et al (2010) Genetic consequences of mass human chemotherapy for *Schistosoma mansoni*: population structure pre- and post-praziquantel treatment in Tanzania. Am J Trop Med Hyg 83: 951–957. 20889898 10.4269/ajtmh.2010.10-0283 PMC2946775

[14] Jones PA, Baylin SB (2007) The Epigenomics of Cancer. Cell 128: 683–692. 17320506 10.1016/j.cell.2007.01.029 PMC3894624

[15] Baylin SB, Jones PA (2011) A decade of exploring the cancer epigenome - biological and translational implications. Nat Rev Cancer 11: 726–734. 21941284 10.1038/nrc3130 PMC3307543

[16] Dawson MA, Kouzarides T (2012) Cancer Epigenetics: From Mechanism to Therapy. Cell 150: 12–27. 22770212 10.1016/j.cell.2012.06.013

[17] Kelly TK, De Carvalho DD, Jones PA (2010) Epigenetic modifications as therapeutic targets. Nat Biotech 28: 1069–1078. 10.1038/nbt.1678 PMC3022972 20944599

[18] Geutjes EJ, Bajpe PK, Bernards R (2011) Targeting the epigenome for treatment of cancer. Oncogene 31: 3827–3844. 22139071 10.1038/onc.2011.552

[19] Huang J, Plass C, Gerhauser C (2011) Cancer chemoprevention by targeting the epigenome. Current Drug Targets 12: 1925–1956. 21158707 10.2174/138945011798184155

[20] Haigentz M Jr, Kim M, Sarta C, Lin J, Keresztes RS, et al (2012) Phase II trial of the histone deacetylase inhibitor romidepsin in patients with recurrent/metastatic head and neck cancer. Oral Oncol 48: 1281–1288. 22748449 10.1016/j.oraloncology.2012.05.024 PMC3465519

[21] Marks PW (2012) Decitabine for acute myeloid leukemia. Exp Rev Anticancer Ther 12: 299–305. 10.1586/era.11.207 22369322

[22] Lyseng-Williamson KA, Yang LPH (2012) Romidepsin: A guide to its clinical use in cutaneous T-cell lymphoma. Am J Clin Dermatol 13: 67–71. 22066664 10.2165/11208520-000000000-00000

[23] Pierce RJ, Dubois-Abdesselem F, Lancelot J, Andrade L, Oliveira G (2012) Targeting schistosome histone modifying enzymes for drug development. Curr Pharm Des 18: 3567–3578. 22607147

[24] Nwaka S, Hudson A (2006) Innovative lead discovery strategies for tropical diseases. Nat Rev Drug Discov 5: 941–955. 17080030 10.1038/nrd2144

[25] Lombardi PM, Cole KE, Dowling DP, Christianson DW (2011) Structure, mechanism, and inhibition of histone deacetylases and related metalloenzymes. Curr Opin Struct Biol 21: 735–743. 21872466 10.1016/j.sbi.2011.08.004 PMC3232309

[26] Atadja PW (2011) HDAC inhibitors and cancer therapy. Prog Drug Res 67: 175–195. 21141730 10.1007/978-3-7643-8989-5_9

[27] Xu WS, Parmigiani RB, Marks PA (2007) Histone deacetylase inhibitors: molecular mechanisms of action. Oncogene 26: 5541–5552. 17694093 10.1038/sj.onc.1210620

[28] Balasubramanian S, Ramos J, Luo W, Sirisawad M, Verner E, et al (2008) A novel histone deacetylase 8 (HDAC8)-specific inhibitor PCI-34051 induces apoptosis in T-cell lymphomas. Leukemia 22: 1026–1034. 18256683 10.1038/leu.2008.9

[29] Bradner JE, West N, Grachan ML, Greenberg EF, Haggarty SJ, et al (2010) Chemical phylogenetics of histone deacetylases. Nat Chem Biol 6: 238–243. 20139990 10.1038/nchembio.313 PMC2822059

[30] Andrews KT, Tran TN, Lucke AJ, Kahnberg P, Le GT, et al (2008) Potent antimalarial activity of histone deacetylase inhibitor analogues. Antimicrob Agents Chemother 52: 1454–1461. 18212103 10.1128/AAC.00757-07 PMC2292526

[31] Mukherjee P, Pradhan A, Shah F, Tekwani BL, Avery MA (2008) Structural insights into the *Plasmodium falciparum* histone deacetylase 1 (PfHDAC-1): A novel target for the development of antimalarial therapy. Bioorg Med Chem 16: 5254–5265. 18362073 10.1016/j.bmc.2008.03.005

[32] Patel V, Mazitschek R, Coleman B, Nguyen C, Urgaonkar S, et al (2009) Identification and characterization of small molecule inhibitors of a class I histone deacetylase from *Plasmodium falciparum* . J Med Chem 52: 2185–2187. 19317450 10.1021/jm801654y PMC2669731

[33] Wheatley NC, Andrews KT, Tran TL, Lucke AJ, Reid RC, et al (2010) Antimalarial histone deacetylase inhibitors containing cinnamate or NSAID components. Bioorg Med Chem Lett 20: 7080–7084. 20951583 10.1016/j.bmcl.2010.09.096

[34] Oger F, Dubois F, Caby S, Noël C, Cornette J, et al (2008) The class I histone deacetylases of the platyhelminth parasite *Schistosoma mansoni* . Biochem Biophys Res Commun 377: 1079–1084. 18977200 10.1016/j.bbrc.2008.10.090

[35] Pierce RJ, Dubois-Abdesselem F, Caby S, Trolet J, Lancelot J, et al (2011) Chromatin regulation in schistosomes and histone modifying enzymes as drug targets. Mem Inst Oswaldo Cruz 106: 794–801. 22124550 10.1590/s0074-02762011000700003

[36] Dubois F, Caby S, Oger F, Cosseau C, Capron M, et al (2009) Histone deacetylase inhibitors induce apoptosis, histone hyperacetylation and up-regulation of gene transcription in *Schistosoma mansoni* . Mol Biochem Parasitol 168: 7–15. 19538992 10.1016/j.molbiopara.2009.06.001

[37] Deardorff MA, Bando M, Nakato R, Watrin E, Itoh T, et al (2012) HDAC8 mutations in Cornelia de Lange syndrome affect the cohesin acetylation cycle. Nature 489: 313–317. 22885700 10.1038/nature11316 PMC3443318

[38] Vannini A, Volpari C, Gallinari P, Jones P, Mattu M, et al (2007) Substrate binding to histone deacetylases as shown by the crystal structure of the HDAC8-substrate complex. EMBO Rep 8: 879–884. 17721440 10.1038/sj.embor.7401047 PMC1973954

[39] Vannini A, Volpari C, Filocamo G, Casavola EC, Brunetti M, et al (2004) Crystal structure of a eukaryotic zinc-dependent histone deacetylase, human HDAC8, complexed with a hydroxamic acid inhibitor. Proc Natl Acad Sci USA 101: 15064–15069. 15477595 10.1073/pnas.0404603101 PMC524051

[40] Watson PJ, Fairall L, Santos GM, Schwabe JWR (2012) Structure of HDAC3 bound to co-repressor and inositol tetraphosphate. Nature 481: 335–340. 22230954 10.1038/nature10728 PMC3272448

[41] Schuetz A, Min J, Allali-Hassani A, Schapira M, Shuen M, et al (2008) Human HDAC7 Harbors a Class IIa Histone Deacetylase-specific Zinc Binding Motif and Cryptic Deacetylase Activity. J Biol Chem 283: 11355–11363. 18285338 10.1074/jbc.M707362200 PMC2431080

[42] Bressi JC, Jennings AJ, Skene R, Wu Y, Melkus R, et al (2010) Exploration of the HDAC2 foot pocket: Synthesis and SAR of substituted N-(2-aminophenyl)benzamides. Bioorg Med Chem Lett 20: 3142–3145. 20392638 10.1016/j.bmcl.2010.03.091

[43] Somoza JR, Skene RJ, Katz BA, Mol C, Ho JD, et al (2004) Structural snapshots of human HDAC8 provide insights into the class I histone deacetylases. Structure 12: 1325–1334. 15242608 10.1016/j.str.2004.04.012

[44] Richon VM, Emiliani S, Verdin E, Webb Y, Breslow R, et al (1998) A class of hybrid polar inducers of transformed cell differentiation inhibits histone deacetylases. Proc Natl Acad Sci USA 95: 3003–3007. 9501205 10.1073/pnas.95.6.3003 PMC19684

[45] Jung M, Brosch G, Kölle D, Scherf H, Gerhäuser C, et al (1999) Amide analogues of trichostatin A as inhibitors of histone deacetylase and inducers of terminal cell differentiation. J Med Chem 42: 4669–4679. 10579829 10.1021/jm991091h

[46] Finnin MS, Donigian JR, Cohen A, Richon VM, Rifkind RA, et al (1999) Structures of a histone deacetylase homologue bound to the TSA and SAHA inhibitors. Nature 401: 188–193. 10490031 10.1038/43710

[47] Nielsen TK, Hildmann C, Dickmanns A, Schwienhorst A, Ficner R (2005) Crystal structure of a bacterial class 2 histone deacetylase homologue. J Mol Biol 354: 107–120. 16242151 10.1016/j.jmb.2005.09.065

[48] Arrowsmith CH, Bountra C, Fish PV, Lee K, Schapira M (2012) Epigenetic protein families: a new frontier for drug discovery. Nat Rev Drug Discov 11: 384–400. 22498752 10.1038/nrd3674

[49] Lane AA, Chabner BA (2009) Histone deacetylase inhibitors in cancer therapy. J Clin Oncol 27: 5459–5468. 19826124 10.1200/JCO.2009.22.1291

[50] Vennervald BJ, Polman K (2009) Helminths and malignancy. Parasite Immunol 31: 686–696. 19825108 10.1111/j.1365-3024.2009.01163.x

[51] Tsai IJ, Zarowiecki M, Holroyd N, Garciarrubio A, Sanchez-Flores A, et al (2013) The genomes of four tapeworm species reveal adaptations to parasitism. Nature 496: 57–63. 23485966 10.1038/nature12031 PMC3964345

[52] Ramalho-Pinto FJ, Gazzinelli G, Howells RE, Mota-Santos TA, Figueiredo EA, et al (1974) *Schistosoma mansoni*: defined system for stepwise transformation of cercaria to schistosomule in vitro. Exp Parasitol 36: 360–372. 4139038 10.1016/0014-4894(74)90076-9

[53] Livak KJ, Schmittgen TD (2001) Analysis of relative gene expression data using real-time quantitative PCR and the 2(-Delta Delta C(T)) Method. Methods 25: 402–408. 11846609 10.1006/meth.2001.1262

[54] Pellegrino J, Siqueira AF (1956) [A perfusion technic for recovery of *Schistosoma mansoni* from experimentally infected guinea pigs]. Rev Bras Malariol Doencas Trop 8: 589–597. 13494879

[55] Diebold ML, Fribourg S, Koch M, Metzger T, Romier C (2011) Deciphering correct strategies for multiprotein complex assembly by co-expression: application to complexes as large as the histone octamer. J Struct Biol 175: 178–188. 21320604 10.1016/j.jsb.2011.02.001

[56] Otwinowski Z, Minor W (1997) Processing of X-ray diffraction data collected in oscillation mode. Methods Enzymol 276: 307–326. 10.1016/S0076-6879(97)76066-X 27754618

[57] Adams PD, Afonine PV, Bunkóczi G, Chen VB, Davis IW, et al (2010) PHENIX: A comprehensive Python-based system for macromolecular structure solution. Acta Crystallogr D Biol Crystallogr 66: 213–221. 20124702 10.1107/S0907444909052925 PMC2815670

[58] Emsley P, Cowtan K (2004) Coot: model-building tools for molecular graphics. Acta Crystallogr D Biol Crystallogr 60: 2126–2132. 15572765 10.1107/S0907444904019158

[59] Emsley P, Lohkamp B, Scott WG, Cowtan K (2010) Features and development of Coot. Acta Crystallogr D Biol Crystallogr 66: 486–501. 20383002 10.1107/S0907444910007493 PMC2852313

[60] Blanc E, Roversi P, Vonrhein C, Flensburg C, Lea SM, et al (2004) Refinement of severely incomplete structures with maximum likelihood in BUSTER-TNT. Acta Crystallogr D Biol Crystallogr 60: 2210–2221. 15572774 10.1107/S0907444904016427

[61] Waterhouse AM, Procter JB, Martin DM, Clamp M, Barton GJ (2009) Jalview Version 2 – a multiple sequence alignment editor and analysis workbench. Bioinformatics 25: 1189–1191. 19151095 10.1093/bioinformatics/btp033 PMC2672624

[62] Wallace AC, Laskowski RA, Thornton JM (1995) LIGPLOT: a program to generate schematic diagrams of protein-ligand interactions. Protein Eng 8: 127–134. 7630882 10.1093/protein/8.2.127

[63] Jones G, Willett P, Glen RC (1995) Molecular recognition of receptor sites using a genetic algorithm with a description of desolvation. J Mol Biol 245: 43–53. 7823319 10.1016/s0022-2836(95)80037-9

[64] Schafer S, Saunders L, Schlimme S, Valkov V, Wagner JM, et al (2009) Pyridylalanine-containing hydroxamic acids as selective HDAC6 inhibitors. ChemMedChem 4: 283–290. 19090524 10.1002/cmdc.200800196

[65] Higa T, Krubsack AJ (1975) Oxidations by thionyl chloride. VI. Mechanism oft he reaction with cinnamic acids. J Org Chem 40: 3037–3045.

[66] Watanabe Y, Iida H, Kibayashi C (1989) Total synthesis of (.+−.)-dihydropinidine, (.+−.)-monomorine I, and (.+−.)-indolizidine 223AB (gephyrotoxin 223AB) by intramolecular nitroso Diels-Alder reaction. J Org Chem 54: 4088–4097.

[67] Stolfa DA, Stefanachi A, Gajer JM, Nebbioso A, Altucci L, et al (2012) Design, synthesis, and biological evaluation of 2-aminobenzanilide derivatives as potent and selective HDAC inhibitors. ChemMedChem 7: 1256–1266. 22628266 10.1002/cmdc.201200193

