# Supplementary figures and images for: Structural Basis for the Inhibition of Histone Deacetylase 8 (HDAC8), a Key Epigenetic Player in the Blood Fluke Schistosoma mansoni
Source: PLoS Pathog. 2013 Sep 26;9(9):e1003645. doi: 10.1371/journal.ppat.1003645 (PMC3784479; doi:10.1371/journal.ppat.1003645)

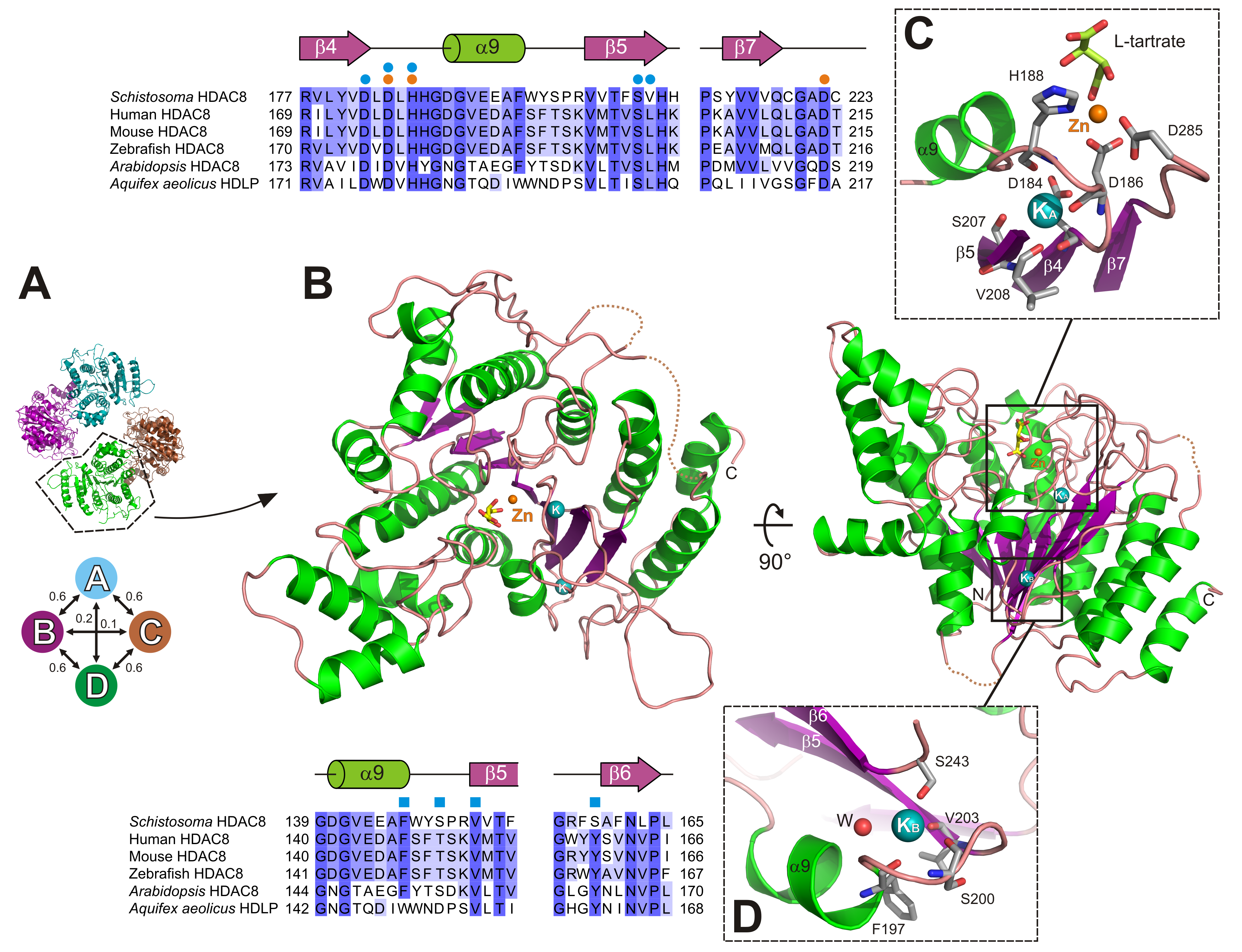

Supplement: Figure S1 — Structural features of smHDAC8. (A) Four non-crystallographic smHDAC8 monomers (A to D) are observed in the asymmetric unit. Root-mean-square deviation (r.m.s.d.) between each monomer range from 0.1 to 0.6 Å. (B) Ribbon representations of the smHDAC8 structure. The L-tartrate molecule bound in the active site is drawn with sticks. Orange sphere, catalytic zinc ion; blue spheres, potassium ions. (C) Close-up view of both the smHDAC8 active site and potassium-binding site A (KA site). Residues involved in zinc and KA binding are drawn in stick representation. The carbon atoms of proteins side chains are in grey, whereas the carbon atoms of L-tartrate are in yellow. The associated multiple alignment shows the conservation of the residues coordinating the zinc ion (orange circles) and the KA ion (blue circles) among HDAC8 family members as well as the HDAC-like bacterial protein HDLP. Note that the tartrate molecule also coordinates the zinc. (D) Close-up view of the potassium binding site B (KB site). Residues involved in the KB ion coordination are drawn in sticks. The red sphere represents a water molecule. The associated alignment shows conservation of residues coordinating the KB ion (blue squares). (TIF) [file ppat.1003645.s001.tif]

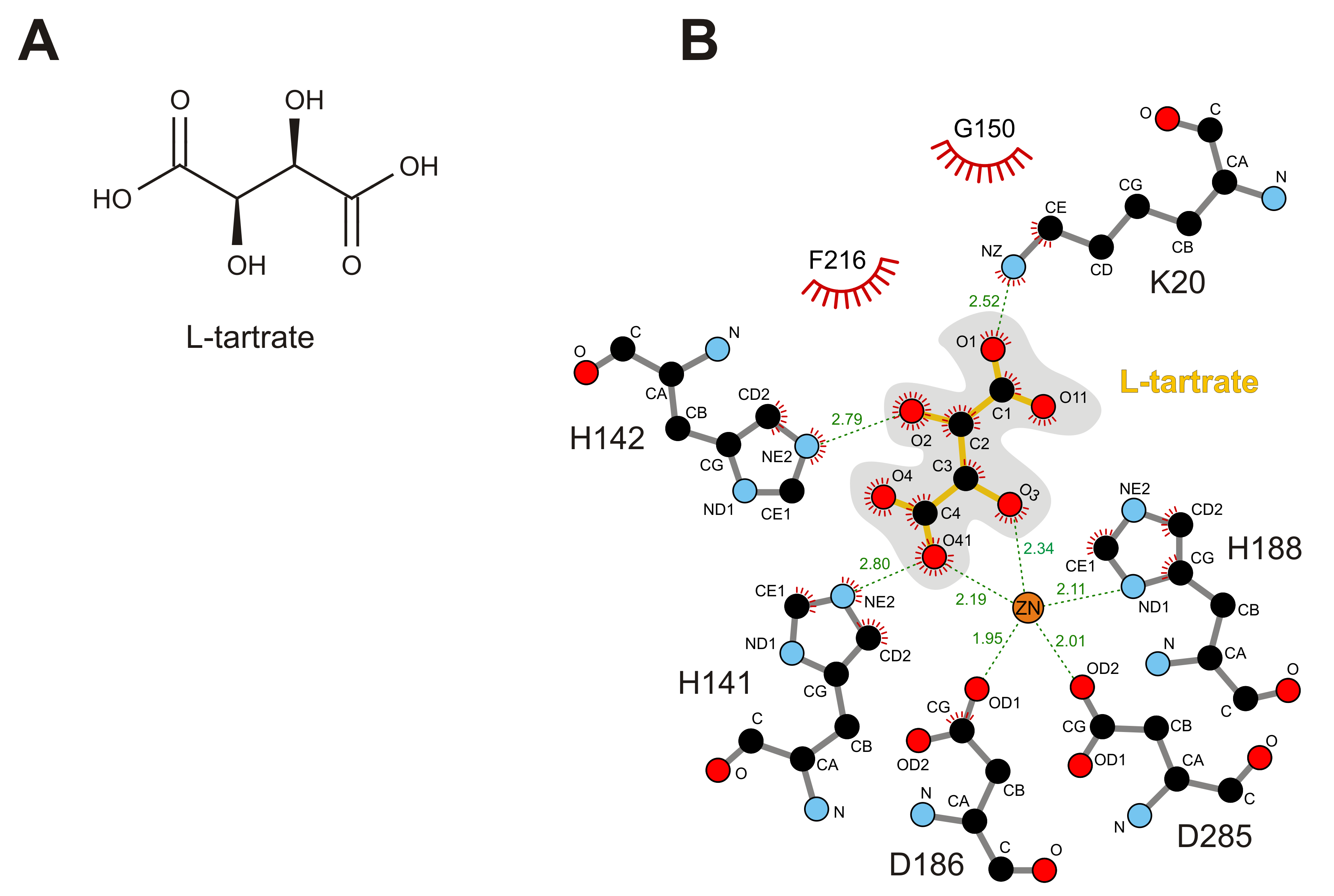

Supplement: Figure S2 — Interaction between native smHDAC8 and the L-tartrate molecule. (A) Chemical structure of L-tartrate molecule. (B) Schematic view of the interactions made by the tartrate molecule as well as the smHDAC8 catalytic zinc ion. The hydrogen bonds and salt bridges observed in the crystal structure are shown with green dotted lines together with their associated distances (Å). Black circles, carbon atoms; red circles, oxygen atoms; blue circles, nitrogen atoms; orange circle, zinc atom. Proteins side chains are in grey, while the L-tartrate scaffold is in yellow. (TIF) [file ppat.1003645.s002.tif]

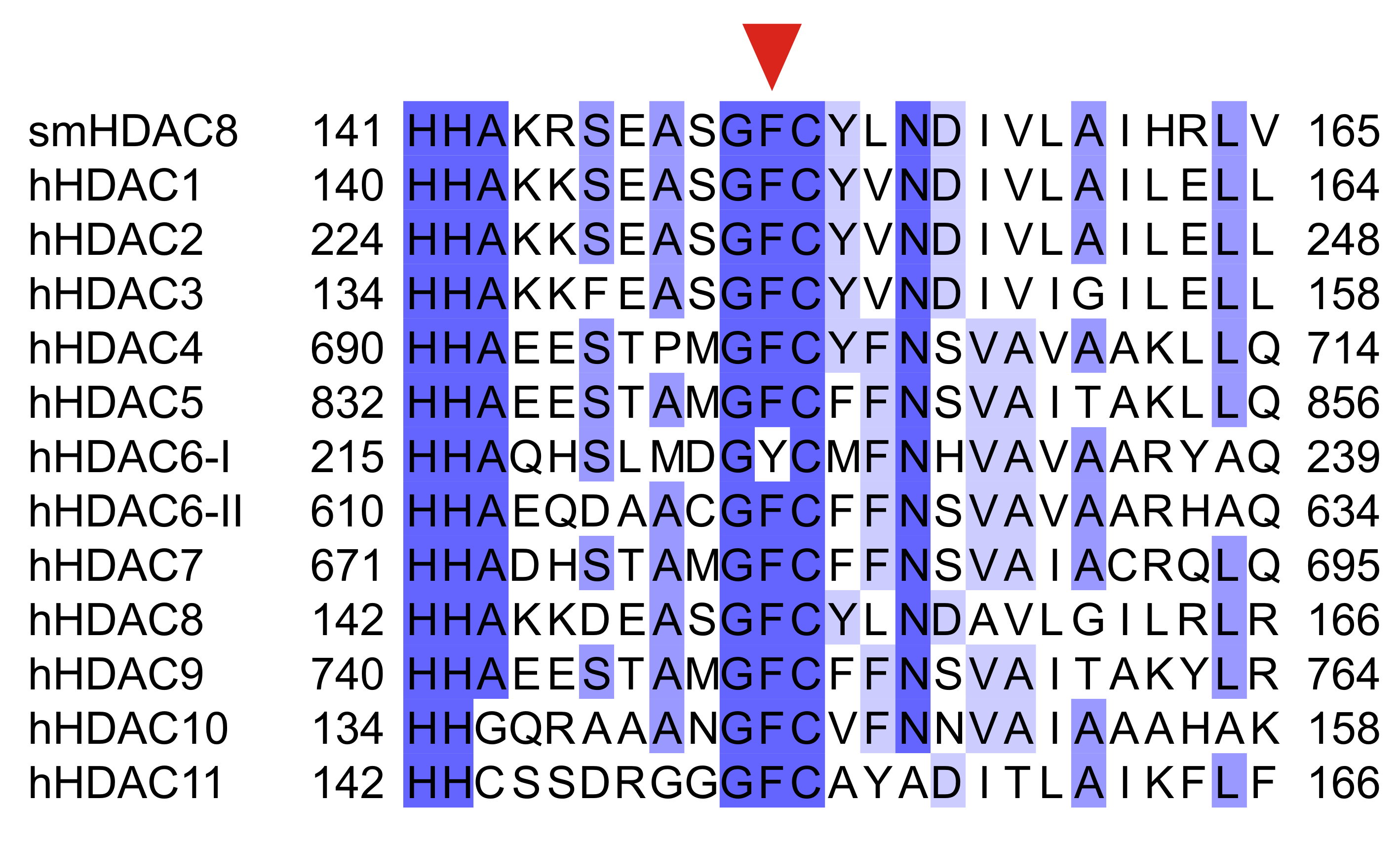

Supplement: Figure S3 — Sequence alignment of smHDAC8 and all human HDACs in the vicinity of the phenylalanine (indicated by a triangle) which adopts either a flipped-in or a flipped-out conformation. Note that hHDAC6 contains two deacetylase domains, which are here designed as hHDAC6-I and hHDAC6-II. The phenylalanine is conserved in all human HDACs, except in the first HDAC domain of hHDAC6 where it is replaced by a tyrosine. Sequence conservation is indicated with blue levels. (TIF) [file ppat.1003645.s003.tif]

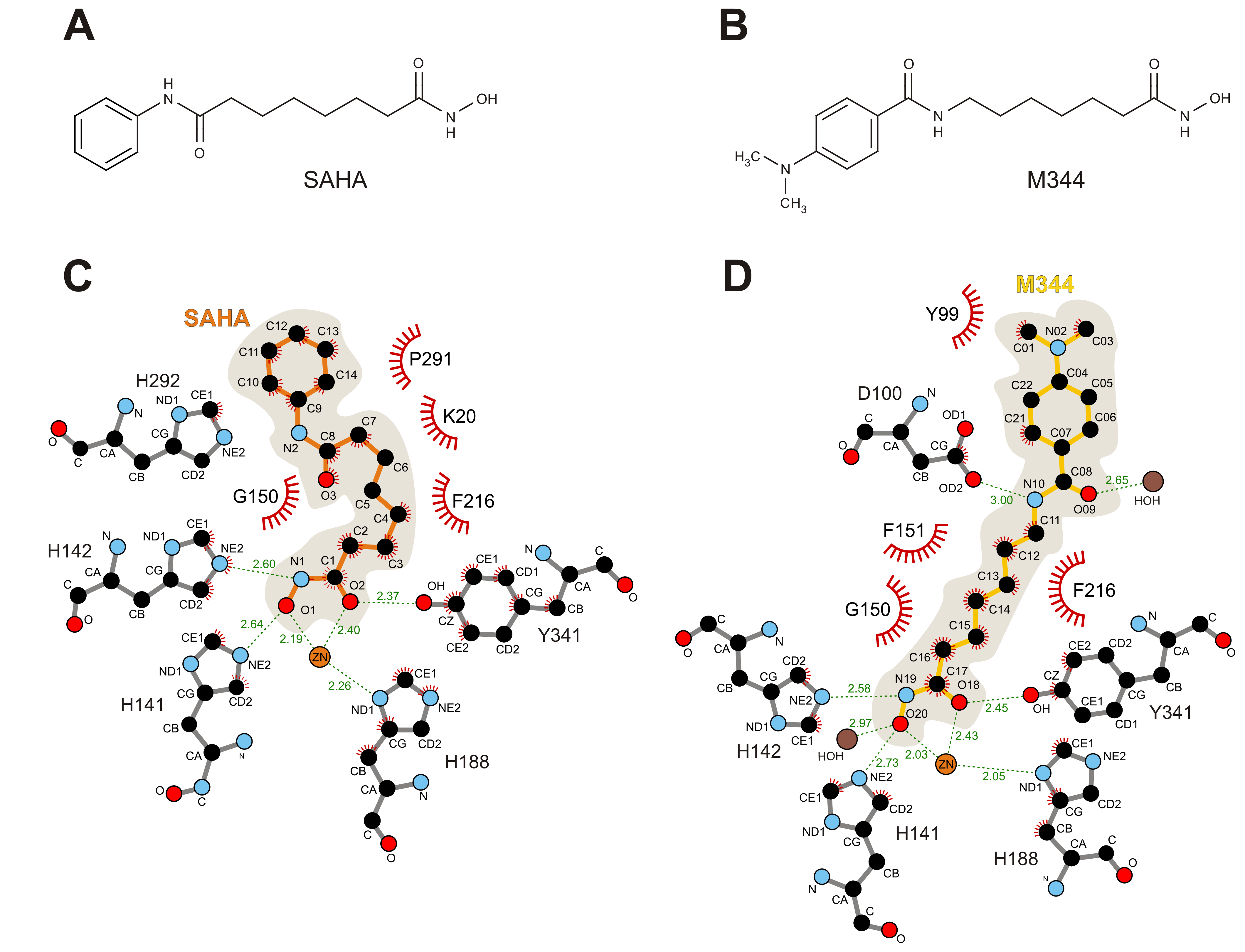

Supplement: Figure S4 — Molecular interactions between smHDAC8 and the generic hydroxamate inhibitors SAHA and M344. (A,B) Chemical structures of SAHA and M344. (C,D) Schematic view of the interactions made by the SAHA and M344 inhibitors with smHDAC8. The same color codes are used than in Figure S2. (TIF) [file ppat.1003645.s004.tif]

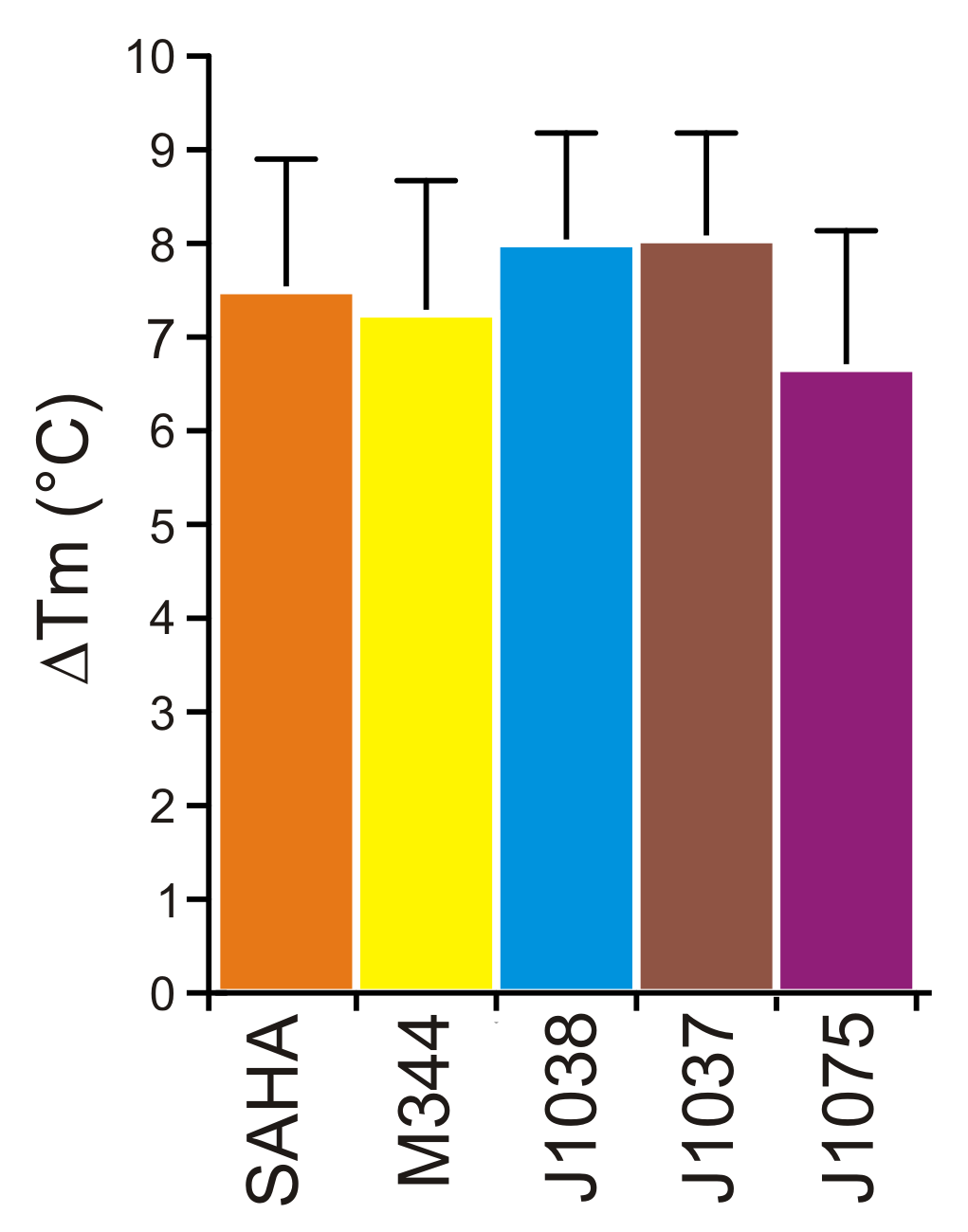

Supplement: Figure S5 — Thermal stabilization of smHDAC8 in presence of the SAHA, M344, J1038, J1037, and J1075 inhibitors. The values represent the mean difference of melting temperatures (ΔTm) observed for smHDAC8 in presence and absence of inhibitors plotted and calculated from three independent assays. Error bars represent the standard deviation (SD). (TIF) [file ppat.1003645.s005.tif]

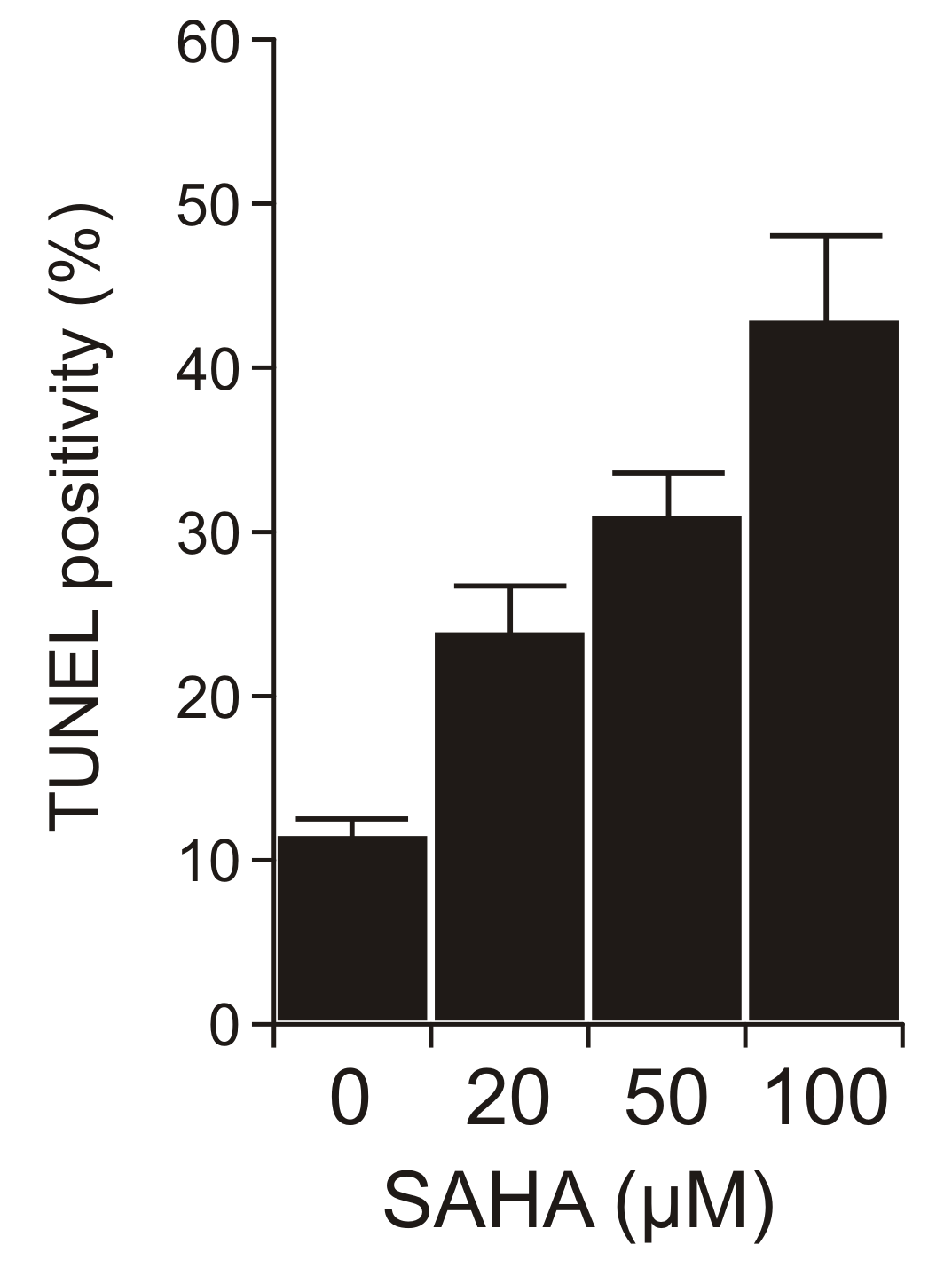

Supplement: Figure S6 — Quantification of TUNEL positivity of schistosomula incubated for 48 h with 0, 20, 50 or 100 µM of SAHA dissolved in DMSO. Results are expressed as the mean values of two independent experiments, with error bars giving the SD. (TIF) [file ppat.1003645.s006.tif]

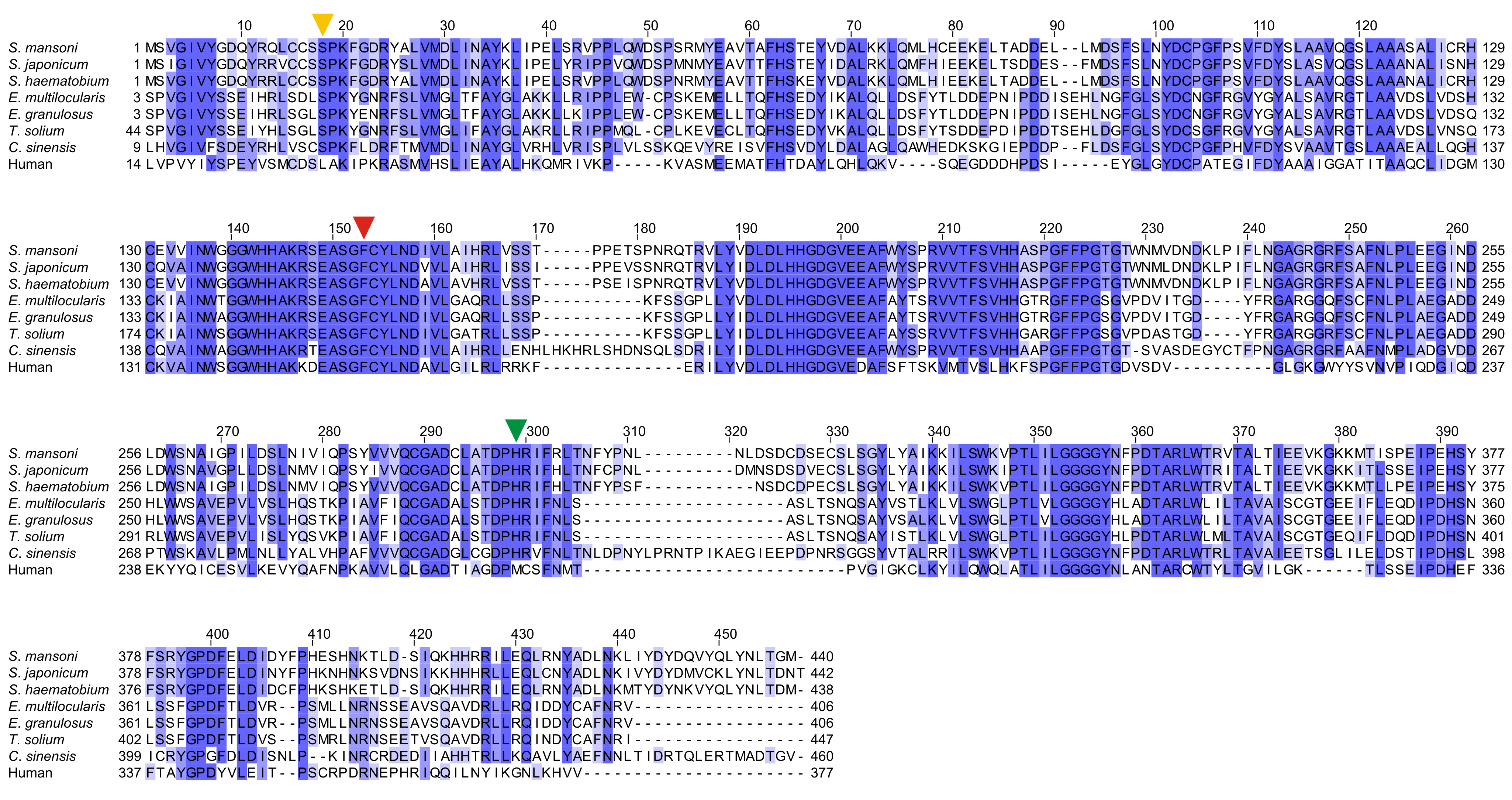

Supplement: Figure S7 — Sequence alignment of schistosome, Echinococcus multilocularis, Echinococcus granulosus, Taenia solium, Clonorchis sinensis and human HDAC8 proteins. Sequences similarities are shown by levels of blue. Important residues that participate in the specificity of Schistosoma mansoni HDAC8 active site and are conserved in the other platyhelminthes HDAC8 proteins are labeled with triangles. The numbering indicated above the alignment corresponds to smHDAC8. (TIF) [file ppat.1003645.s007.tif]
